# Approaches in Gene Coexpression Analysis in Eukaryotes

**DOI:** 10.3390/biology11071019

**Published:** 2022-07-06

**Authors:** Vasileios L. Zogopoulos, Georgia Saxami, Apostolos Malatras, Konstantinos Papadopoulos, Ioanna Tsotra, Vassiliki A. Iconomidou, Ioannis Michalopoulos

**Affiliations:** 1 Centre of Systems Biology, Biomedical Research Foundation, Academy of Athens, 11527 Athens, Greece; vzogopoulos@bioacademy.gr (V.L.Z.); gsaxami@hua.gr (G.S.); kopap@biol.uoa.gr (K.P.); itsotra@biol.uoa.gr (I.T.); 2 Section of Cell Biology and Biophysics, Department of Biology, National and Kapodistrian University of Athens, 15701 Athens, Greece; veconom@biol.uoa.gr; 3 Department of Nutrition and Dietetics, Harokopio University, 17671 Athens, Greece; 4 Biobank.cy Center of Excellence in Biobanking and Biomedical Research, University of Cyprus, 2029 Nicosia, Cyprus; malatras.apostolos@ucy.ac.cy

**Keywords:** gene coexpression networks, transcriptomics, RNA-Seq, microarrays, systems biology, webtool

## Abstract

**Simple Summary:**

Genes whose expression levels rise and fall similarly in a large set of samples, may be considered coexpressed. Gene coexpression analysis refers to the en masse discovery of coexpressed genes from a large variety of transcriptomic experiments. The type of biological networks that studies gene coexpression, known as Gene Coexpression Networks, consist of an undirected graph depicting genes and their coexpression relationships. Coexpressed genes are clustered in smaller subnetworks, the predominant biological roles of which can be determined through enrichment analysis. By studying well-annotated gene partners, the attribution of new roles to genes of unknown function or assumption for participation in common metabolic pathways can be achieved, through a guilt-by-association approach. In this review, we present key issues in gene coexpression analysis, as well as the most popular tools that perform it.

**Abstract:**

Gene coexpression analysis constitutes a widely used practice for gene partner identification and gene function prediction, consisting of many intricate procedures. The analysis begins with the collection of primary transcriptomic data and their preprocessing, continues with the calculation of the similarity between genes based on their expression values in the selected sample dataset and results in the construction and visualisation of a gene coexpression network (GCN) and its evaluation using biological term enrichment analysis. As gene coexpression analysis has been studied extensively, we present most parts of the methodology in a clear manner and the reasoning behind the selection of some of the techniques. In this review, we offer a comprehensive and comprehensible account of the steps required for performing a complete gene coexpression analysis in eukaryotic organisms. We comment on the use of RNA-Seq vs. microarrays, as well as the best practices for GCN construction. Furthermore, we recount the most popular webtools and standalone applications performing gene coexpression analysis, with details on their methods, features and outputs.

## 1. Introduction

The development of high-throughput technologies [1] aided the discovery of biological networks which provide insights into the understanding of system properties [2,3,4]. An earlier classification [5] divided biological networks into four groups:

- Protein–protein interaction (PPI) networks [6] describe the associations, either through physical contact or common pathway participation, between two or more proteins;
- Gene regulatory networks (GRNs) [7] depict the causal interactions between regulators and their target genes;
- Signal transduction networks [8] contain information on the interactions between biochemical signalling molecules and cell receptors;
- Metabolic and biochemical networks [9] display all metabolic reactions and molecules involved in biological pathways.

Due to the recent accumulation of large amounts of transcriptomic data through microarray and RNA-Seq technologies, an additional group of biological networks has emerged [10,11]: Gene coexpression networks (GCNs) [12] allow the study of the coexpression patterns of multiple genes in different biological conditions.

Gene coexpression networks depict the degree of similarity between the expression profiles of all genes, in a particular set of biological samples that may derive from different tissues, developmental stages, or environmental conditions, to reach conclusions far beyond the scopes of the individual studies the samples have come from. The underlying basis of gene coexpression analysis is that coexpressed genes tend to participate in similar biological processes [13,14]. Furthermore, expression levels of correlated genes may be controlled by similar regulatory mechanisms. As such, GCNs can replicate known functional roles and regulatory interactions between genes. The construction of GCNs can additionally function as a prediction method, identifying novel functional interactions between genes, as well as assigning new roles to existing genes or genes of yet unknown function [15,16].

Currently, there are several gene coexpression webtools and standalone applications focusing on a variety of model species of animals [13,17,18,19,20,21], plants [22,23,24,25,26,27,28,29] and fungi [30,31].

Many methods have been developed for the construction of a gene coexpression network [12,32]. However, most of the methodologies include the following steps:

1. Collection and integration of expression data
2. Processing and filtering of gene expression data and construction of expression matrices [12,24]
3. Selection of coexpression measure and construction of similarity matrices [15,32]
4. Selection of significance threshold and network construction [24,33].
5. Identification of modules using clustering techniques [32].

We review key issues in the analysis of gene coexpression and the basic features for the construction of a GCN. In addition, the most popular gene coexpression applications for various model organisms, are presented.

## 2. Collection and Processing of Transcriptomic Data and Construction of Gene Expression Matrices

The two main transcriptomic technologies used to obtain expression data for coexpression analysis are microarrays [34] and RNA-Seq [35]. The samples used for a coexpression analysis can be procured from public databases, produced through in-house experiments by research groups, or a combination of both. Using publicly available experiments is usually preferred, as many public transcriptomic data repositories provide an abundance of expression profiling studies. The most popular ones include Gene Expression Omnibus (GEO) [36], ArrayExpress [37], and Expression Atlas [38] which contain both microarray and RNA-Seq data, as well as Sequence Read Archive (SRA) [39], Gene-Tissue Expression (GTEx) [40], The Cancer Genome Atlas (TCGA) [41] and European Nucleotide Archive (ENA) [42], which are RNA-Seq specific.

The source data must originate from the same organism and the same transcriptomic platform for the coexpression results to be comparable. Subsequently, there are two major approaches to coexpression analysis, depending on the experimental conditions of the primary sample data sets used [3]:

(A) ‘Condition independent’ approach uses a set of samples of a multitude of different conditions and source tissues. This method is suitable for studying the global coexpression landscape of an organism and demonstrates gene relationships regardless of experimental conditions [12].
(B) ‘Condition dependent’ [12,43] approach uses a set of samples that derive from a specific tissue or a specific experimental condition. In this case, the coexpression analysis aims to discover the gene coexpression profile under the selected condition.

The biological question at hand defines which one of the two approaches should be adopted. Since all aforementioned transcriptomic data repositories describe in detail each of their available samples and can be queried using integrated advanced search functions, samples of the same species from the same platform can be easily retrieved. This sample filtering strategy can be expanded to identify samples of a specific tissue or condition.

Another important point lies in the total number of samples used for the coexpression analysis. Although using a small number of samples results in stronger gene correlations, it also increases the chance for spurious correlations to appear [3]. Consequently, a minimum amount of 20 samples is recommended to perform a coexpression analysis [44].

### 2.1. Microarray Data Analysis

**Figure 1:**
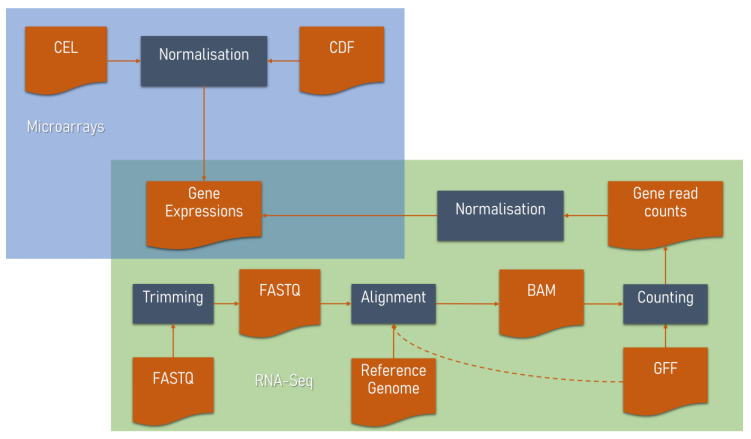
Pre-processing procedure for transcriptomic data. Primary microarray data are procured in a CEL format which is transformed to gene expression values by using a normalisation algorithm which is guided by a Chip Description File (CDF). In RNA-Seq primary data pre-processing, the FASTQ-formatted sequence read data are trimmed, then aligned to a reference genome. Gene counts are produced with the help of a General Feature Format (GFF) file. GFF file may also be used during alignment. Expression values are produced through normalisation. Both technologies eventually converge to the production of the same output, an expression matrix which contains the expressions of each gene in all samples.

There are several microarray manufacturers, such as Affymetrix [45], Agilent [46], Illumina [47], etc. Among them, Affymetrix GeneChip is the most popular platform to quantify gene expression. For each Affymetrix microarray hybridisation, a CEL file that contains the intensity values per probe is produced. Those primary files are then pre-processed with the assistance of a Chip Description File (CDF) which describes probe locations and probe set groupings on the chip, to calculate the expression values per probe set. These values are combined with an annotation file that contains gene-probe set correspondences, to obtain the gene expression values (Figure 1). Microarray pre-processing algorithms, usually referred to as normalisation algorithms, include the following steps:

1. background correction
2. normalisation
3. probe summarisation
4. log_2_ transformation (optional)

The most popular normalisation methods that lead to one expression value per probe set are MAS5 [48], RMA [49], GCRMA [50], PLIER [51] and SCAN [52]. The oldest of these algorithms, MAS5, is the only one that does not perform logarithm transformation to the expression values. SCAN and MAS5 algorithms normalise each microarray sample independently of the others of the same series and are preferred when combining microarray samples from different series or laboratories, as other pre-processing algorithms, such as RMA or GCRMA, derive information from all samples together during normalisation and thus potentially introduce erroneous calculations, known as correlation artifacts. To eliminate low-quality samples [53], a great effort has been made to develop methods to assess and visualise the quality of GeneChip data. Specific algorithms for quality control (QC) have been developed and many of these have been implemented in R statistical scripting language [54] and are available in Bioconductor suite [55].

### 2.2. RNA-Seq Data Analysis

Since its introduction, RNA-Seq has been steadily increasing as the method of choice to measure gene expressions accurately. The RNA-Seq technology that studies the aggregated mRNA of cell populations or tissue parts is also referred as bulk RNA-Seq. RNA-Seq is based on next-generation sequencing (NGS) where the length of the reads does not exceed 700 bps [56] and third-generation sequencing where the read length can be more than 150,000 bps [57]. Next-generation sequencing technologies include Illumina [58], 454 Life Science [59], etc, while third-generation sequencers include PacBio [60], Nanopore [61], etc. The raw data produced by RNA-Seq experiments are FASTQ [62] files, containing the sequence reads, as well as a quality value for each base. The pre-processing of RNA-Seq data [63] consists of:

1. quality control and trimming of sequence reads
2. mapping reads to a reference genome or transcriptome
3. producing gene read counts
4. normalisation

The first step of the pre-processing pipeline includes the quality assessment of the sequence reads and subsequent trimming of the adapter sequences and low-quality reads [64]. Software for quality control includes FastQC [65] which produces per-sample reports and MultiQC [66] which aggregates these reports, producing a single summary report and LongQC [67] which is specific for third-generation sequencing data. Software for trimming includes Cutadapt [68], fastp [69] and Trimmomatic [70]. Complete removal, also known as hard-clipping, is usually performed exclusively on the adapter sequences to save up storage space and facilitate downstream analysis. Soft-clipping refers to tagging low-quality reads or adapter sequences, so that they can be ignored in later steps of the analysis. Soft-clipping is preferrable to hard-clipping, as important information regarding the reads is not completely lost. Next, the trimmed reads are aligned to FASTA-formatted sequences of their corresponding reference genome. This step is performed using specific alignment software depending on the sequence read length: Aligners such as TopHat2 [71] and HISAT2 [72] are used for short reads, Magic-Blast [73], Graphmap2 [74], DART [75] LAMSA [75] and deSALT [76] for long reads and Bowtie 2 [77], minimap2 [78], STAR [79] GMAP [80] and BWA-MEM [81] for both types of reads. Some aligners can also perform soft-clipping of bases from the left or right end of the read sequence [79] and unmapped reads will always be soft-clipped during the alignment step. This process produces a BAM-formatted [82] file which contains the mapping of the reads to the reference genome. This output is then combined with a General Feature Format 3 (GFF3) file [83] which contains the genomic feature coordinates, to count the gene reads, using programs such as Cufflinks [84], featureCounts [85] and HTSeq [86]. Aligners may also use GFF3 annotations upfront. The exon joints provided by GFF files, accelerate the mapping process and increase the quality of the spliced alignments. Finally, to calculate the gene expression values, the resulting gene read count data are normalised. Algorithms such as Total Count [87], Quantile [88] and Upper Quartile [89], are purely based on arithmetic calculations concerning the read counts and their distributions in the samples, while TPM [90] and RPKM [91] take transcript length into account. TMM [92] and DESeq [93] use a mathematical and biological combination and qsmooth [94] normalises read counts based on the assumption that the distribution of samples should differ on a global scale, but not in each biological group/tissue. After normalisation, log_2_ transformation of expression data is applied (Figure 1). Other software, such as Kallisto [95] and Salmon [96], use a different approach, pseudoaligning reads to a reference transcriptome, producing gene expression data two orders of magnitude faster than other pipelines. The selection of the normalisation algorithm impacts the quality of the resulting GCNs [97], thus, different normalisation procedures might be chosen for condition-independent or condition-dependent analyses.

### 2.3. Single-Cell RNA-Seq in Coexpression Analysis

Single-cell RNA-Seq (scRNA-Seq) is a recently emerging RNA-Seq-based technology which studies the transcriptome of single cells [98]. The pre-processing pipeline of scRNA-Seq data is similar to that of bulk RNA-Seq data. However there are certain additional steps that need to be performed, to account for the high heterogeneity of single-cell data [99]. A common phenomenon in scRNA-Seq data, is the appearance of a large amount of zero counts of genes that are truly expressed in other cells of the same type, known as dropout events [100]. In order to fill in the missing values, imputation methods, such as scImpute [101], SAVER [102] and MAGIC [103], have been developed. The produced expression matrix includes the expression values of genes per sample which in this case refers to a single cell.

### 2.4. Microarrays vs. RNA-Seq in Coexpression Analysis

The end result of both microarray and RNA-Seq data pre-processing is a file containing gene expression values per sample. Affymetrix-based chips use an outdated default CDF, so several probe sets either do not correspond to any known gene or correspond to more than one genes, and some genes are recognised by no probe set or by more than one probe sets. Thus, a custom CDF that better reflects current genomic and transcriptomic knowledge is recommended. One such example is the frequently updated BrainArray CDF [104] which ensures that each probe set corresponds to a single gene and vice versa.

RNA-Seq is a rapidly evolving technology with a larger, ever-increasing amount of publicly available data. As opposed to microarrays, RNA-Seq can accurately measure all known genes of an organism and has higher sensitivity. However, the expression estimations of RNA-Seq and microarrays are comparable, especially in genes with average expression [105]. Thus, the resulting gene coexpression landscapes which derive from RNA-Seq and microarrays are close [106] and biological pathway enrichments are similar [22]. The drawbacks of RNA-Seq include the significantly longer execution time of data pre-processing and higher computational resource requirements, as well as the use of pipelines of not yet fully optimised algorithms. On the contrary, all steps in microarray pre-processing are performed by a single, quick, light and optimised algorithm (Figure 1).

Irrespective of the transcriptomic technology, pre-processing of existing raw transcriptomic data from public repositories is imperative, as it ensures data uniformity which is essential for subsequent coexpression analysis. Reanalysis of the original primary data with modern normalisation algorithms and genomic annotations, can highly improve the estimation of gene expressions and thus, the coexpression landscape. This is crucial, especially in the case of microarray data analysis, as it was reported that up to 50% of the genes that were identified as differentially expressed in Affymetrix-based studies where default CDF was used, might be artifacts [104].

### 2.5. Batch Correction

There are many conditions which may vary during the course of an experiment (such as reagents, equipment, personnel, etc.) and may introduce batch effects, which is a common source of variation in both microarray and RNA-Seq data [107]. In the case of condition dependent (tissue-specific) coexpression analysis where data from multiple studies are combined, another layer of batch effects is introduced: experiments from different laboratories. Thus, batch effect identification and subsequent correction is an important step after expression data pre-processing. Usually, the studies that each sample belongs to, are used to define the batches, although the date and time of each experiment may be used as batch surrogates. Existence of batch effects is confirmed through visual inspection via principal component analysis (PCA) [108] and hierarchical clustering [109]. Batch effects are present if samples from the same study which derive from different biological conditions are clustered together, whereas the clusters should have been made up of the samples of the same conditions, regardless of study source. Batch-corrected microarray-based coexpression analysis using ComBat [110], produces combined correlations which are more consistent with each single study’s correlations [106], while a larger number of high quality GCNs are produced when ComBat batch correction is applied to normalised RNA-Seq data [97]. While ComBat requires manual denoting of the sources of the batch effects, SVA [111] can automatically estimate them, and subsequently applies ComBat correction. SVA is useful in cases where there are indications of technical variations (e.g., observed by PCA) but their source is not evident. scRNA-Seq samples are much more prone to technical variations, due to the low amount of genetic material isolated from each cell [99]. In this case, batch effect correction is perfomed by scRNA-Seq specific methods, such as f-scLVM [112], MNN [113] and kBET [114].

## 3. Selection of Coexpression Measure and Construction of Similarity Matrices

After the acquisition of gene expression data, the correlation of expression between each gene pair needs to be calculated. This is performed through a vast variety of approaches:

Distance-based measures calculate the dissimilarity between the expression of a pair of genes. Traditional distance measures are based on Minkowski distances [115]:

$$d_{min}={\left(\sum_{i=1}^{n}{\left|x_{i-}y_{i}\right|}^{m}\right)}^{\frac{1}{m}}$$

where *m* is a positive integer and $x_{i}$ and $y_{i}$ are the expression values of *x* and *y* genes in the *i*th sample. Euclidean and Manhattan distances are cases of Minkowski distance, depending on the value of *m*. In Manhattan distance, *m* = 1:

$$d=\sum_{i=1}^{n}\left|x_{i-}y_{i}\right|$$

In one of the most used distance measures, Euclidean distance, *m* = 2:

$$d=\sqrt{\sum_{i=1}^{n}{\left(x_{i-}y_{i}\right)}^{2}}$$

Correlation metrics describe the tendency of the expression levels of a pair of genes, to increase or decrease simultaneously across different samples [3,4]. They produce coefficients ranging from −1 (perfect anti-correlation) to +1 (perfect correlation), with values near 0 indicating no correlation.

The Pearson correlation coefficient (PCC or *r*) [116] is a measure that depicts the linear correlation between two genes, *x* and *y*, and is calculated as follows:

$$r=\frac{\sum_{i=1}^{n}\left(x_{i}-\bar{x}\right)\left(y_{i}-\bar{y}\right)}{\sqrt{\sum_{i=1}^{n}{\left(x_{i}-\bar{x}\right)}^{2}\sum_{i=1}^{n}{\left(y_{i}-\bar{y}\right)}^{2}}}$$

where *n* is the number of samples and *x_i_* and *y_i_* are the expression values of *x* and *y* genes in the *i*th sample. PCC is useful for detecting correlation between genes that may have different average expression levels, however in some cases it is sensitive to outliers [3,12] resulting in false-positive results when the number of samples is small and pre-processing is based on quantile normalisation [117].

Uncentred correlation (Cosine similarity) [118] depicts the similarity between the expression of two gene pairs and, in contrast to centred PCC, it does not take into account the mean expression of each gene. It is given by:

$$cos_{sim}\left(x,y\right)=\frac{\sum_{i=1}^{n}x_{i}y_{i}}{\sqrt{\sum_{i=1}^{n}{\left(x_{i}\right)}^{2}}\sqrt{\sum_{i=1}^{n}{\left(y_{i}\right)}^{2}}}$$

Spearman’s rank correlation coefficient (*ρ*) [119] is calculated as the PCC of the rankings of the expression values. In cases where there are no ranking ties, *ρ* can be calculated as follows [120]:

$$\rho \left(x,y\right)=1-\frac{6\sum_{j=1}^{n}D_{j}^{2}}{n\left(n^{2}-1\right)}$$

where *D_j_* is the difference between the ranks of the corresponding values of genes x and y.

As a parametric measure, PCC is used if gene expression values follow normal distributions across samples, otherwise a nonparametric method, such as Spearman’s rank correlation coefficient, should be used. The selection of the algorithm can be based on a normality test. As Spearman’s correlation coefficient uses expression ranks instead of expression values, *ρ* is less sensitive to extreme data values.

Kendall’s rank correlation coefficient (*τ*) [121] is a measure of nonlinear dependence between two random variables. It is suitable for identifying key genes that increase or decline in monotonic fashions in expression data collected during a biological process or developmental stage [122]. For any pair of observations $\left\{\left(x_{i},x_{j}\right),\;\left(y_{i},y_{j}\right)\right\}$ of expressions of genes *x* and *y* in samples *i* and *j*, where *i* < *j*, if (*x_i_ > x_j_* AND *y_i_ > y_j_*) OR (*x_i_ < x_j_* AND *y_i_ < y_j_*), the pair is concordant, if (*x_i_ > x_j_* AND *y_i_ < y_j_*) OR (*x _i_*< *x_j_* AND *y_i_ > y_j_*) the pair is discordant, or if *x_i_ = x_j_* OR *y_i_ = y_j_*, the pair is neither concordant nor discordant. Kendall’s correlation coefficient is given by [122]:

$$\tau =\frac{n_{c}-n_{d}}{\sqrt{\left[\frac{n\left(n-1\right)}{2}-\sum_{k}\frac{t_{k}\left(t_{k}-1\right)}{2}]|\frac{n\left(n-1\right)}{2}-\sum_{l}\frac{u_{l}\left(u_{l}-1\right)}{2}\right|}}$$

where *n* is the number of samples, *n_c_* is the number of concordant observation pairs, *n_d_* the number of discordant pairs, $t_{k}$ is the number of observations tied at *k* rank of *x* and $u_{l}$ is the number of observations tied at *l* rank of *y*. In cases where there are no tied observations, the following formula is used:

$$\tau =\frac{n_{c}-n_{d}}{\frac{n\left(n-1\right)}{2}}$$

Since Kendall’s rank correlation coefficient is used to identify monotonic relationships, it is used as an alternative to Spearman’s.

The aforementioned correlation coefficient values are used to compute the Mutual Rank (MR) [123] score as follows:

$$M_{xy}=\sqrt{R_{xy}R_{yx}}$$

where *R_xy_* is the rank of the correlation of genes *x* and *y* in the descending list of all gene correlations of *x*. Since MR is a distance measure, with smaller values meaning higher correlation, a Logit Score (LS) transformation [124] is applied:

$$L_{xy}=\log_{2}\left(N-M_{xy}\right)-\log_{2}\left(M_{xy}\right)$$

where *N* is the total number of genes studied. Higher values of LS indicate stronger correlations.

Finally, Mutual Information (MI) is a method that detects the amount of information obtained about the expression of one gene by observing the expression of another gene [125]. MI is based on Shannon’s theory of communication [126] and is calculated by subtracting the joint entropy of two genes *X* and *Y* from the sum of their entropies [127]:

$$I\left(X,Y\right)=H\left(X\right)+H\left(Y\right)-H\left(X,Y\right)$$

A MI value which is close to 0 surmises no correlation between a gene pair, while a high value shows a correlation relationship. In contrast to PCC, Mutual information can detect non-linear statistical relationships [128].

## 4. Selection of Significance Thresholds for Network Construction

Once a correlation measure has been chosen, a correlation matrix which contains all pairwise gene correlation coefficients $cor\left(x,y\right)$ for any *x* and *y* genes, is constructed. The correlation matrix is a square matrix with M × M dimensions, where M is the number of studied genes. The diagonal values of the matrix are 1, as they correspond to the correlation of any gene to itself and the matrix is symmetric to the main diagonal, thus it can also be portrayed as an upper or lower triangular matrix, displaying each gene pair correlation once.

**Figure 2:**
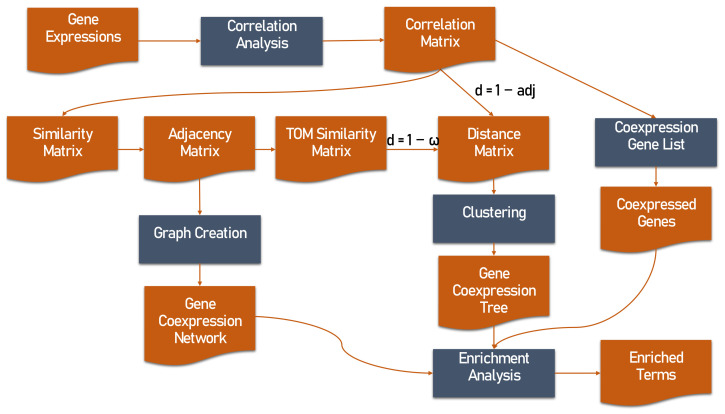
Flowchart depicting the steps for performing gene coexpression analysis using gene expression data. Gene pairwise correlations are calculated and regardless of the chosen correlation measure, correlation values need to be transformed to similarity values and then to adjacency values. Gene coexpression can be depicted as lists, dendrograms or networks. Eventually, the results of the coexpression analysis need to be evaluated through enrichment analysis.

There are several ways to portray the correlation landscape of a large number of genes (Figure 2). The simplest and commonest way to study gene coexpression, is by producing a list of most coexpressed genes to a “driver gene” i.e., the gene of interest. In this coexpressed gene list [129], the correlations of the driver gene with all other genes are ranked according to their correlation coefficient, either in descending order to highlight the top positively correlated genes, or in ascending order to highlight the top negatively correlated genes. In effect, a coexpression list contains the ordered values of the correlation matrix row (or column) of the driver gene, thus it demonstrates singular gene coexpression relationships, without accounting for any interconnections among the coexpressed genes of the list.

To overcome the aforementioned limitation, a more sophisticated way to study gene coexpression is the construction of a GCN, based on an M × M similarity matrix which scales all correlation values between 0 and 1. If the absolute correlation values are used for the construction of the matrix ($s_{xy}=\left|cor\left(x,y\right)\right|$, where $s_{xy}\;$ is the similarity between *x* and *y* genes), then the similarity matrix is considered “unsigned”. In unsigned similarity matrices, positively and negatively correlated gene pairs cannot be distinguished. To tackle this, “signed” similarity matrices are produced as follows:

$$s_{xy}=\frac{1+cor\left(x,y\right)}{2}$$

with negative correlations getting $s_{xy}$ < 0.5 and positive correlations getting $s_{xy}$ ≥ 0.5 [130].

An adjacency value $a_{xy}$ between genes *x* and *y* is produced by applying an adjacency function to the similarity values ($s_{xy}$). Depending on the function used, a certain type of significance threshold is applied to reveal significant biological relationships. Threshold selection can be divided into hard and soft threshold approaches. Hard thresholds exclude gene pairs with similarity values below the predetermined threshold τ [131] by mapping all similarity values to 0 or 1 adjacency values, to show the absence or presence of coexpression between a pair of genes:

$$a_{xy}=\left\{\begin{array}{c} 1\;if\;s_{xy}\;\geq \tau \;\\ 0\;if\;s_{xy}<\tau \end{array}\right.$$

Another hard threshold approach is to set the adjacency values of only a certain top percent of the similarity values, to 1 [132]:

$$a_{xy}=\left\{\begin{array}{c} 1\;if\;s_{xy}\;\geq P_{r}\;\\ 0\;if\;s_{xy}<P_{r} \end{array}\right.$$

where $P_{r}$ is the *r*th percentile of all *s* values (i.e., *r*% of *s* values are less than or equal to $P_{r}$).

In graph theory, GCNs are depicted as a set of vertices (nodes) which correspond to genes and undirected edges (lines connecting node pairs) which represent gene pair correlations [3,12,133,134]. Unweighted networks can be produced only if gene pairs of adjacency values equal to 1 are drawn as edges. To avoid self-loops, the values of the main diagonal of the binary adjacency matrix are set to 0. The most popular program to visualise GCNs is Cytoscape [135] which is also available as a web plugin [136].

When adjacency values are produced using soft thresholds, similarity values are transformed through specific functions resulting in adjacency values which range between 0 and 1 [130]. If the power function is selected, the adjacency value is calculated as follows:

$$a_{xy}={\left|s_{xy}\right|}^{\beta }$$

where *β* is a parameter chosen by the user. Soft thresholds result in weighted networks, where each weight is used to appraise the strength of the coexpression relationship. Weighted networks depict all available coexpression relationships between each gene pair with each edge being coupled with a corresponding weight value. To avoid accidental noise and incorrect correlations, the transformation of adjacency matrix into Topological Overlap Measure (TOM) matrix is proposed [130]. A TOM matrix displays the strength of connection between two genes *x* and *y* (Farber & Mesner, 2016) and is calculated as follows:

$$\omega_{xy}=\frac{\sum_{u\neq x,y}a_{xu}a_{uy}+a_{xy}}{min\left(\sum_{u\neq x}a_{xu},\sum_{u\neq y}a_{yu}\right)+1-a_{xy}}$$

where $\omega_{xy}$ is the TOM similarity value, *u* is a gene other than *x* and *y* and $a_{xy}$ is the adjacency value of *x* and *y*.

A distance or dissimilarity matrix contains the distance values between each gene pair. A distance matrix from a correlation matrix can be produced by applying a $d=1-cor\left(x,y\right)$ transformation [137] to all correlation values. As such, a distance matrix has the same M×M dimensions, is symmetric to its main diagonal and can be displayed as an upper or lower triangular matrix. The values range from 0 (complete correlation) to 2 (complete anti-correlation), with values around 1 showing no correlation. The diagonal values of the distance matrix are 0, as they correspond to the distance of a gene to itself. The TOM matrix can also be transformed into a distance matrix by subtracting its values from 1 [130]:

$$d_{xy}^{\omega }=1-\omega_{xy}$$

Even though both hard and soft thresholds result in GCNs, it is not easy to select a cut-off value to achieve the optimal connections in a network. An extremely high cut-off may fail to reveal important relationships, missing crucial biological information, while a generous one will result in spurious relationships [138].

## 5. Identification of Modules Using Clustering Techniques

Modules in a GCN can be defined as a group of genes that are densely linked [139,140,141]. Highly connected genes within a network are called hub genes. These genes have been shown to be functionally significant [142,143]. There are two types of hub genes named intra-modular and inter-modular hubs that are central to specific modules in the network or central to the entire network, respectively [32].

Clustering is a method to group and visualise coexpressed genes, using a distance matrix as input. Genes that have similar expression patterns across multiple samples are grouped to produce sets of coexpressed genes [32,125]. The most common clustering method is hierarchical clustering whose most popular implementation in gene coexpression is the unweighted pair group method with arithmetic mean (UPGMA) [109]. Hierarchical clustering starts by connecting genes that are closest to each other and continues to connect resulting clusters based on their pairwise distances, eventually forming a tree (in this case, a gene coexpression tree). The leaves of the tree represent the genes and the lengths of the branches reflect the distance between genes, thus tree clades represent coexpression modules [32,125,137]. The tree output file is usually in Newick format [144].

Biclustering generates clusters of rows and columns simultaneously [145]. In the case of gene expression, rows are genes and columns are samples. Biclustering is usually depicted in the form of a coexpression heatmap. Based on their expression level, genes are mapped into clusters with the main objective to find homogeneous submatrices called biclusters which may overlap, or discover local expression patterns according to certain experimental conditions [146]. Due to this process, biological information about these clusters can be extracted. This information refers not only to the correlated genes but also to the identification of genes that do not act the same way in all conditions [147].

A popular non-hierarchical clustering method is *k*-means, a partitioning method that subdivides the genes into a predefined *k* number of clusters [137,148]. The *k*-means method initially sets *k* points that function as cluster centre points (centroids). Each gene is then assigned to the cluster with the closest centroid. New positions for the cluster centroids are set as the average of the genes of the cluster, and gene assignment begins anew. The previous two steps continue until no more genes change cluster [137,149]. However, it is difficult to determine the optimal number of *k* points and multiple runs of the algorithm may result in different components for each cluster.

The self-organizing map (SOM) method is closely related to *k*-means, also starting with a predetermined number of cluster centroids. In the case of SOMs though, the centroids are linked in a prespecified geometrical configuration [149]. Each iteration involves randomly selecting a gene and moving the closest centroid in the direction of this gene, as well as its neighbouring centroids on the grid [137]. In this fashion, neighbouring centroids in the initial geometry tend to be mapped to nearby centroids in *k*-dimensional space [150]. Clusters that are closest to each other in the initial arrangement, tend to be more similar to each other than those that are further apart [149]. The end result is a grid of clusters, in which neighbouring clusters show related expression patterns [137].

Gene coexpression trees produced through clustering cannot portray anti-coexpressed genes and are limited to classifying a gene into a single functional cluster, although genes may possess multiple functions and participate in different metabolic pathways [23].

The cophenetic correlation coefficient (CPCC or *c*) is used to measure the quality of hierarchical clustering [151]. Cophenetic pairwise distances are calculated as the pairwise distances between genes as these are portrayed by the gene coexpression tree. CPCC is the PCC between the initial pairwise distance of genes and their cophenetic pairwise distance [152]:

$$c=\frac{\sum_{x}^{n}\left(d_{xy}-\bar{d}\right)\left(t_{xy}-\bar{t}\right)}{\sqrt{\sum_{x}^{n}{\left(d_{xy}-\bar{d}\right)}^{2}\sum_{x}^{n}{\left(t_{xy}-\bar{t}\right)}^{2}}}$$

where $d_{xy}$ is the distance of genes *x* and *y* from the original distance matrix and $t_{xy}$ is their cophenetic distance. CPCC shows how faithfully the coexpression tree has retained the initial pairwise distances and ranges from −1 to 1, with 0 surmising no relation at all and 1 showing that the dendrogram has perfectly replicated the distances between genes.

## 6. Gene List Functional Enrichment Analysis

The purpose of a gene coexpression analysis is to discover functional gene partners to a gene of interest. Biological functions can be attributed to genes of unknown role, based on the verified functions of their coexpressed gene partners [12], an approach known as “guilt by association”. By identifying the most coexpressed genes to a gene of interest or the subnetwork or subtree that the gene of interest belongs to, from a GCN or a gene coexpression tree, respectively, lists of highly coexpressed genes are created. The predominant biological functions, metabolic pathways, regulating transcription factors, disease associations, etc, for such a gene list can be determined through functional enrichment analysis.

In over-representation analysis (ORA), statistically significant biological terms which describe members of a list of coexpressed genes, are discovered by comparing the observed number of genes of the list which are related to a certain biological term, against the expected number of genes which would be related to the same term. Thus, a reference list containing all the studied genes, as well as sufficient biological annotations, is required. The statistical significance of enriched terms is usually assessed by calculating *p*-values through Fisher’s exact test or the hypergeometric distribution test [153].

In gene set enrichment analysis (GSEA) [154], all genes are ordered according to their correlation values with the gene of interest, with the top and bottom extremes being the top most coexpressed and top anti-coexpressed genes, respectively. Already compiled gene sets of several biological categories are used as background gene libraries. The enrichment score (ES) for a biological term is calculated as follows: A running-sum value of the ranked list of coexpressed genes is computed, increasing every time a gene that appears in the gene library is found, and decreasing otherwise. Its maximum observed value becomes the ES of that specific biological term. By generating a null distribution for the ES through permutation, the statistical significance is estimated by calculating a *p*-value.

Enrichment analysis *p*-values need to be adjusted for multiple comparisons. This is done by calculating the false discovery rate (FDR) [155]. In the case of GSEA, a normalised enrichment score (NES) is first calculated before producing the FDR adjusted *p*-value. Statistically significant terms have an adjusted *p*-value below a predetermined cut-off. The lower the adjusted *p*-value of the biological term, the more confident we are of the term truly being enriched.

Biological term enrichment categories include: gene ontologies [156], biological and metabolic pathways [157], protein structures [158], gene-disease associations [159], regulatory motifs [160], experimentally verified transcription factor binding sites [161], etc. Public online tools performing enrichment analysis of coexpressed gene lists that result from coexpression analyses include g:Profiler [162], Enrichr [163], WebGestalt [164], FLAME [165], DAVID [166] and GOnet [167]. More specifically, g:Profiler offers enrichment analyses for more than 700 organisms. FLAME can perform many visualisations on the input gene list but its enrichment analysis is based on g:Profiler calculations. Enrichr offers an immense list of available biological term compilations, but is available only for six model species. Compared to the other tools, DAVID and WebGestalt can be used with or without a reference gene list, with WebGestalt allowing for detailed parameter customisation before analysis. Most of the tools also offer integrated functions for gene ID conversions. Finally, GOnet can perform gene ontology enrichment analysis only for human and mouse, but is unique in visualising the input genes and their corresponding enriched gene ontologies as well as the ontology hierarchy and relationships between ontologies as a graph.

## 7. Coexpression Tools

Several coexpression tools studying global or tissue-specific coexpression analysis, are available at the time of writing, as online websites (Table S1), or as stand-alone applications. We are presenting a brief description of the main functionalities of each tool as well as emphasising their distinguishing features.

### 7.1. Global Coexpression Web Tools

**Figure 3:**
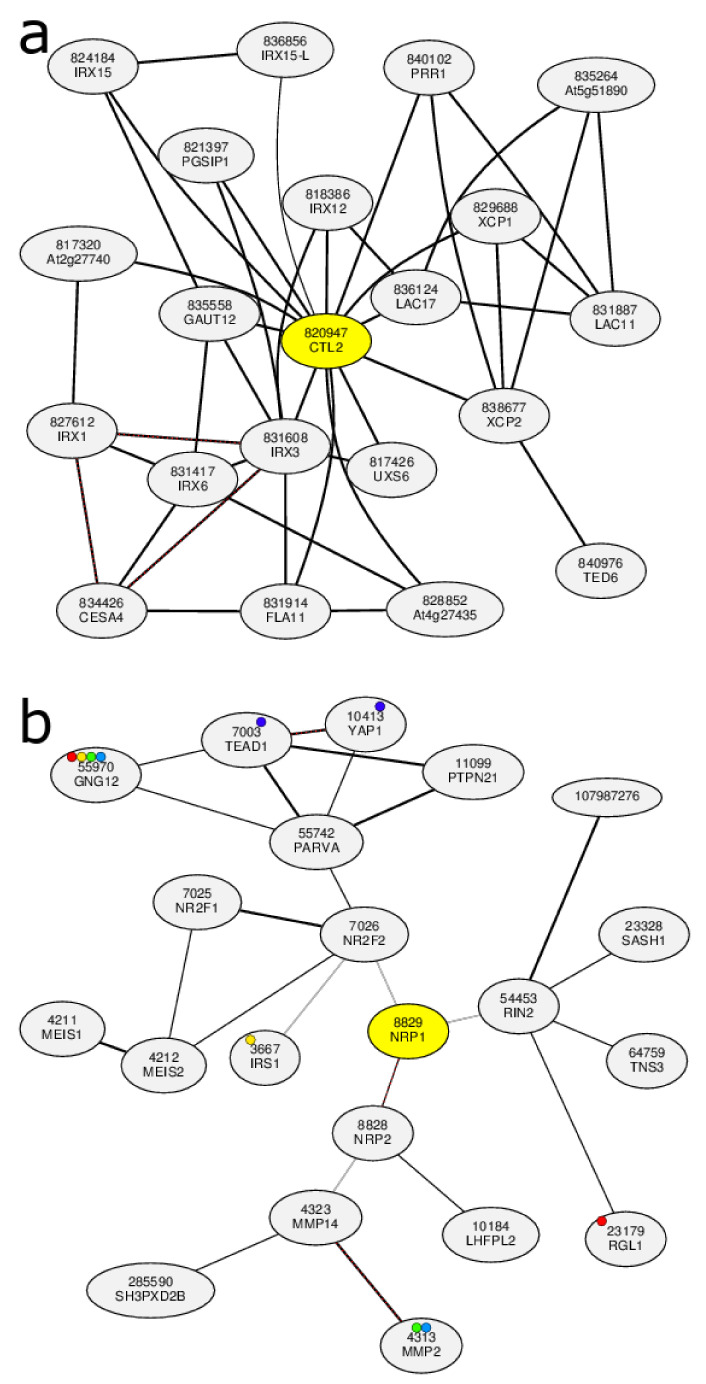
Coexpression results of ATTED-II and COXPRESdb: (**a**) GCN of the top coexpressed partners to *CTL2*, found in the gene’s information page; (**b**) GCN of the top coexpressed gene partners to *NRP1*, found in the gene’s information page. Coloured circles refer to different KEGG pathways.

**COXPRESdb** [168] provides gene coexpression relationships, for nine animal and two fungal species: *Homo sapiens*, *Mus musculus*, *Rattus norvegicus*, *Gallus gallus*, *Macaca mulatta*, *Canis lupus familiaris*, *Danio rerio*, *Drosophila melanogaster*, *Caenorhabditis elegans*, *Saccharomyces cerevisiae* and *Schizosaccharomyces pombe*. **ATTED-II** [124] is the sister database to COXPRESdb, providing coexpression data for nine plant species: *Arabidopsis thaliana*, *Brassica rapa*, *Glycine max*, *Medicago truncatula*, *Oryza sativa*, *Populus trichocarpa*, *Solanum lycopersicum*, *Vitis vinifera* and *Zea mays*. COXPRESdb and ATTED-II contain both microarray and RNA-Seq data and are constantly evolving with new features and increasing numbers of samples. The databases use the Logit Score transformed mutual ranks as a gene coexpression measure and RNA-Seq data are processed with their own Matataki [169] quantification software, an algorithm optimised for execution speed. The coexpression results are portrayed as coexpressed gene lists, sorted in descending LS order of coexpressed genes with the gene of interest, based on representative gene expression data combining both RNA-Seq and microarrays. Adjacent lists display results from all other available transcriptomic subsets, such as microarray samples from specific conditions, etc. Furthermore, to increase the robustness of the analysis, coexpression results of orthologous genes of closely related species are also displayed. Finally, the top coexpressed partners to a gene of interest are portrayed as coexpression networks in the gene’s information page (Figure 3).

**Figure 4:**
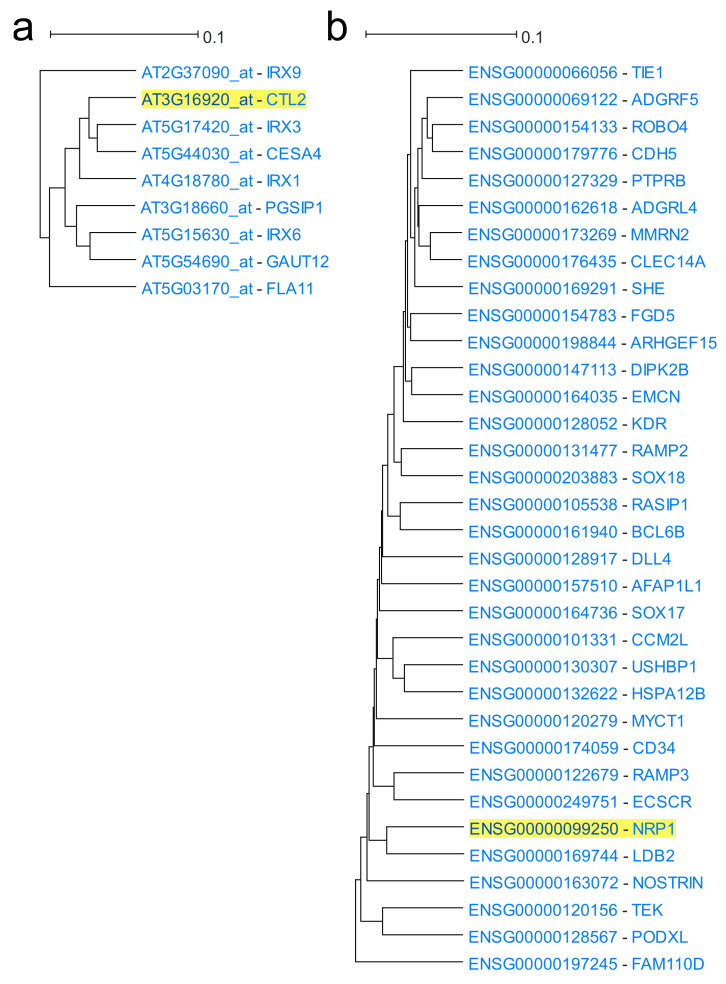
Coexpression results of ACT and HGCA2.0: (**a**) Default coexpression subtree in ACT using *CTL2* as driver gene. The subtree contains nine genes (including the driver gene) and possesses five ancestral nodes; (**b**) Default coexpression subtree in HGCA2.0 using *NRP1* as driver gene. The subtree contains 34 genes (including the driver gene) and possesses five ancestral nodes.

**Arabidopsis Coexpression Tool (ACT)** [23,140,141] studies gene coexpression in 21,273 *Arabidopsis thaliana* genes using high-quality healthy microarray samples. The latest version of ACT is based on 3500 Affymetrix Arabidopsis ATH1 Genome Array GeneChip samples from ArrayExpress, GEO and NASCArrays. Expression data were produced using the SCAN algorithm along with Brainarray CDF. Genes were clustered using UPGMA hierarchical clustering to create a gene coexpression tree. Using a single gene as input, a subclade containing the driver gene and its coexpressed genes is produced (Figure 4a). The subtree size can be increased or decreased. Multiple biological term enrichment analyses are offered and the coexpression subtree and its corresponding gene list can be exported to various external tools for further downstream analysis. ACT’s sister web tool for *Homo sapiens* is **Human Gene Correlation Analysis (HGCA)** [13]. HGCA1.0 is based on 1959 Affymetrix Human Genome U133 Plus 2.0 samples of various cells and tissues. Gene expression data were produced using the MAS5.0 algorithm with default CDF. Pairwise PCCs were measured for all probe sets and were grouped using neighbour joining [170]. Similar to ACT, users select a driver probe set which corresponds to the gene of interest. Users can choose between two outputs: a coexpressed gene list or a gene coexpression tree. Over-representation analysis for multiple biological categories is also available. HGCA1.5 is based on the same samples as HGCA1.0. Nevertheless, primary data are processed in a manner identical to that of ACT. HGCA2.0 is a major upgrade as expression data from 55,431 genes were produced from GTEx RNA-Seq gene count data of 3500 samples, using qsmooth normalisation. The downstream data processing is similar to that of HGCA1.5. HGCA1.5 and HGCA2.0 output gene coexpression trees (Figure 4b).

**EXPath 2.0** [171] allows the user to perform various transcriptomic-based analyses for six plant species: *Arabidopsis thaliana*, *Oryza sativa*, *Zea mays*, *Solanum lycopersicum*, *Glycine max*, and *Medicago truncatula*. EXPath 2.0 contains both microarray and RNA-Seq data from various conditions. Single gene analysis in EXPath 2.0 has multiple outputs: EXPath offers information for a gene of interest, including its biological terms, sample-specific expression and top correlated or anti-correlated genes. A multiple gene query results in a weighted GCN that includes both positively and negatively coexpressed genes. Finally, GO and pathway enrichment, as well as differential expression gene analyses are available.

**PLAΝt coEXpression (PLANEX)** [28] is a coexpression database for eight plant species: *Arabidopsis thaliana*, *Glycine max*, *Hordeum vulgare*, *Oryza sativa*, *Solanum lycopersicum*, *Triticum aestivum*, *Vitis vinifera* and *Zea mays*. This database presents a list of coexpressed genes ranked by their PCCs. Positive and negative cut-offs were determined by finding the top 1% of the positive and the top 1% of the negatively correlated gene pairs. Furthermore, a GCN can also be presented. Another functionality is the comparison of the coexpression between any user-selected gene pair. Compared with other similar databases, in PLANEX’s case the probes were mapped against representative genes by string match instead of BLAST [172], thus producing positive results if each base in a probe sequence matched perfectly with the representative gene sequence without any gap. In addition, the PCC was subjected to PCA, for the identification of a gene set with changing expression over different experiments.

**Co-expressed biological Processes (CoP)** [173] is a microarray-based database for eight model or popular plant species: *Arabidopsis thaliana*, *Glycine max*, *Hordeum vulgare, Oryza sativa*, *Populus trichocarpa*, *Triticum aestivum*, *Vitis vinifera* and *Zea mays*. For a gene and species of interest, CoP outputs the following: Gene details, coexpressed gene list of the driver gene’s coexpression module, homologous genes in the same species, orthologous genes in the other seven plant species included in CoP, as well as the microarray experiments where it is explicitly expressed. Gene correlations are calculated using uncentred correlation coefficients and coexpressed gene modules are determined through the confeito algorithm [174]. Each coexpression module is associated with biological processes and metabolic pathways.

**Figure 5:**
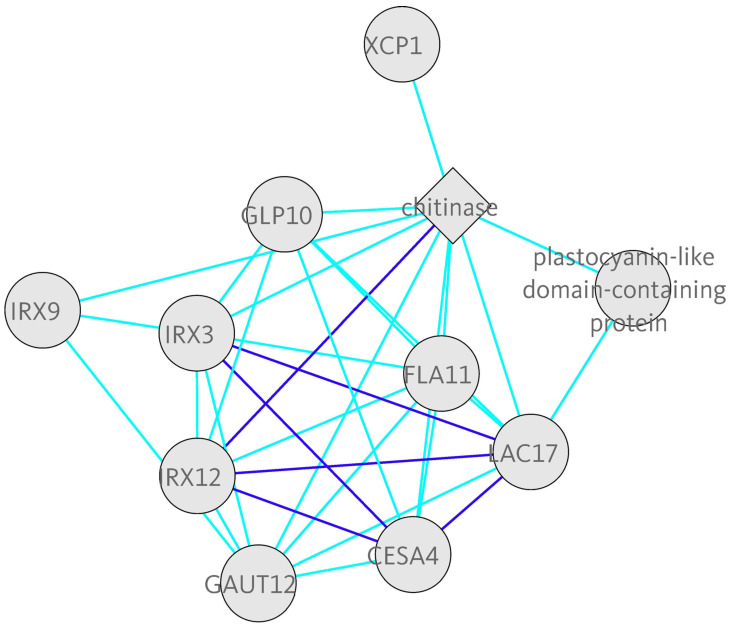
GCN of ten coexpressed partners to *CTL2* in CorNet, visualised through Cytoscape. The GCN includes the coexpression inter-relationships.

**Correlation Networks (CorNet)** [175] is an online tool for network construction in *Arabidopsis thaliana*. CorNet is based on microarray and RNA-Seq samples and can perform coexpression, protein–protein or regulatory interaction analyses. Using pre-defined or user-uploaded primary datasets, CorNet displays the coexpressed genes to a single gene or a list of genes. Various customisation options are available: selecting between Pearson or Spearman correlation coefficients and setting a correlation threshold, *p*-value cut-off, the number of resulting coexpressed genes and whether the GCN will contain relationships between the coexpressed genes. The output is either a GCN which is visualised through Cytoscape (Figure 5) or a coexpressed gene list.

**Mouse Gene Prediction Database** [176] is one of the first databases studying gene coexpression in *Mus musculus*. It is based on custom-made Agilent microarray samples, using custom-mapped probes. The user can input a mouse gene of interest and the web tool outputs a coexpression intensity heatmap which contains the top 100 probe sets (as well as their corresponding genes) which are coexpressed with the gene of interest on one axis and the available tissues on the other axis. It is also possible to search for groups of coexpressed genes associated with a GO term or to search for genes via their genomic location or sequence similarity through BLAST.

**Figure 6:**
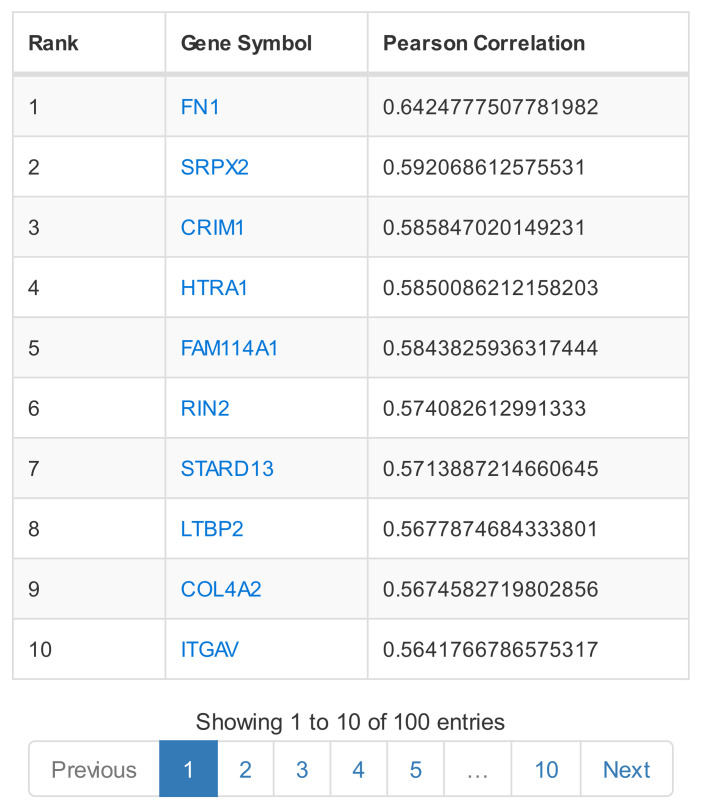
Coexpression gene list in ARCHS^4^. The full list corresponds to the top 100 coexpressed genes to *NRP1*, with only the top ten being presented.

**ARCHS^4^** [21] is based on RNA-Seq gene count data for *Homo sapiens* and *Mus musculus*, derived from GEO and SRA samples. The web tool displays a scatter cloud 3d-visualisation of all genes based on their coexpression similarity. Single gene search outputs the predicted biological functions of the gene of interest, specific tissue/cell-line gene expression, as well as a list of the top coexpressed genes (Figure 6). Enrichment analysis on the list of coexpressed genes can be performed through Enrichr.

**Search-based Exploration of Expression Kompendia (SEEK)** [177] includes thousands of microarray and RNA-Seq samples which are used for gene coexpression analysis in *Homo sapiens*. Both single and multiple gene searches are available: in the single gene search, coexpressed genes to the gene of interest are displayed, starting from the top coexpressed ones. The coexpression score of each gene is calculated across the selected datasets. By using the “expression” option, each gene’s specific expression in each sample is displayed as a heatmap. Each dataset produces a different expression heatmap and samples belonging to the same dataset are grouped through hierarchical clustering. By using the “co-expression” option, a single heatmap containing the summarized coexpression scores across 50 datasets at a time, is displayed. The sample datasets can be filtered to include specific tissues or cell-lines. Multiple gene query has a similar output, with the addition of an extra heatmap showing the sample-specific expression among the query genes. Enrichment analysis, including metabolic pathways, gene ontology categories, etc., is available.

**Figure 7:**
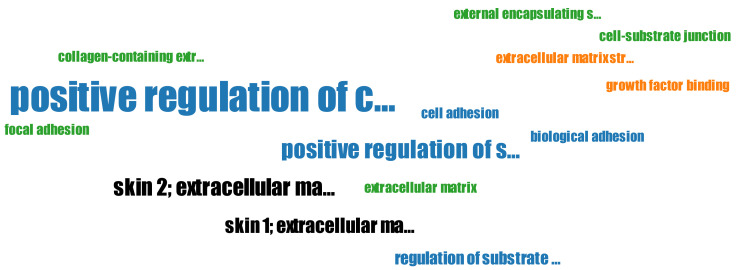
Enrichment analysis results depicted as a word cloud produced by MEM. The resulting biological terms are derived using the top 50 coexpressed genes to *NRP1* in MEM. Some terms and names may be clipped. Nevertheless, full term names can be found in an accompanying table below the word cloud in the MEM webpage.

**Multi Experiment Matrix (MEM)** [178] allows analysis of multiple transcriptomic datasets derived from ArrayExpress and GEO for *Homo sapiens*, *Mus musculus*, *Arabidopsis thaliana*, *Rattus norvegicus*, *Saccharomyces cerevisiae*, *Drosophila melanogaster*, *Sus scrofa*, *Oryza sativa*, *Escherichia coli*, *Danio rerio*, *Caenorhabditis elegans*, *Gallus gallus*, *Bos taurus*, *Pseudomonas aeruginosa*, *Medicago truncatula*, *Triticum aestivum*, *Macaca mulatta*, *Canis familiaris*, *Populus trichocarpa*, *Hordeum vulgare*, *Zea mays*, *Glycine max*, *Staphylococcus aureus*, *Xenopus laevis*, *Solanum lycopersicum*, *Vitis vinifera*, *Anopheles gambiae* and *Xenopus tropicalis*. Users can perform coexpression analysis for one or multiple genes in the experiments of each available platform. Using a single gene as query, results in a gene expression heatmap, with the top coexpressed genes on one axis and the selected experiments grouped through hierarchical clustering on the other axis. Gene ontology enrichment analysis can be performed with the results also being portrayed as a word cloud (Figure 7). MEM allows for a lot of customisations, such as using multiple different correlation measures and filtering the resulting coexpressed genes.

**Gemma** [179] performs differential gene expression or coexpression analysis for single or multiple genes in a user-selected sample dataset. The following species are included: *Homo sapiens*, *Mus musculus*, *Rattus norvegicus*, *Drosophila melanogaster*, *Saccharomyces cerevisiae*, *Danio rerio* and *Escherichia coli*. By selecting a single gene, a list of the top coexpressed genes is produced. A heatmap of the expression of each coexpressed gene pair can be displayed in each available series of samples. Other gene details, such as sample datasets where the gene is differentially expressed, or the disease phenotypes associated with the gene of interest, are also included. A multiple gene coexpression analysis can be performed, either by inputting a custom gene list or selecting from already compiled gene lists. The transcriptomic samples to be used in the analysis can also be selected from Gemma’s database. A multiple gene coexpression analysis produces a list of positively or negatively coexpressed genes that can also be visualised as a GCN. Selected nodes in the resulting GCN can be expanded with additional correlated genes.

**Search Tool for the Retrieval of Interacting Genes/Proteins (STRING)** [180] is a popular web tool performing PPI network construction for 12,025 Bacteria, 1597 Eukaryotes and 472 Archaea. STRING accepts a gene or a gene list as input and outputs an expanded PPI network, containing the input genes’ corresponding proteins as well as additional predicted protein interactors. A GCN can be constructed by only selecting “Co-expression” from all interaction sources available. An important feature of STRING is “Analysis” which includes multiple enrichment analyses calculated in-house, as well as important network statistics, such as average local clustering coefficient and PPI enrichment *p*-value.

**Figure 8:**
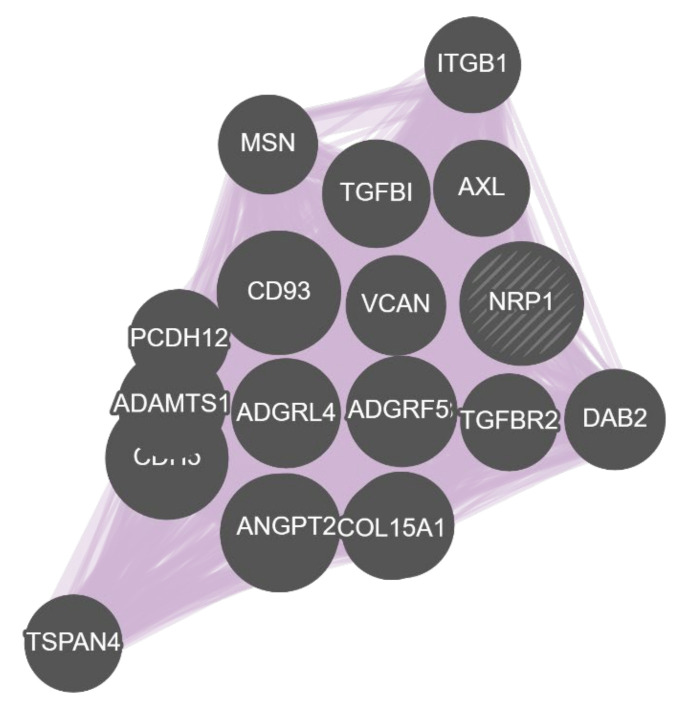
GeneMANIA-produced GCN using *Homo sapiens NRP1* as driver gene. Only coexpression relationships were used, with the rest of the settings being the default ones.

**Gene Multiple Association Network Integration Algorithm (GeneMANIA)** [31] is a web tool for gene network construction and function prediction for *Homo sapiens*, *Mus musculus*, *Rattus norvegicus*, *Drosophila melanogaster*, *Saccharomyces cerevisiae*, *Danio rerio*, *Escherichia coli*, *Arabidopsis thaliana* and *Caenorhabditis elegans*. GeneMANIA accepts either a single gene or a list of genes as input and outputs a gene network depicting multiple gene–gene relationships including coexpression and protein interactions [181,182]. By selecting only coexpression-based evidence relationships, a GCN is effectively created. If a single gene is selected as input, GeneMANIA outputs a user preselected amount of coexpressed gene partners (default 20) to the gene of interest (Figure 8). All data associated with the generated GCN, such as the Cytoscape network file, the gene list of the GCN or the list of publications supporting the relationships between the coexpressed genes can be downloaded.

### 7.2. Condition-Specific Coexpression Web Tools

**GeneFriends** [183] offers tissue-specific gene coexpression networks for 20 human and 21 mouse tissues as well as global gene coexpression networks for *Homo sapiens*, *Mus musculus*, *Bos taurus*, *Rattus norvegicus*, *Danio rerio*, *Drosophila melanogaster*, *Gallus gallus* and *Saccharomyces cerevisiae*. The latest version is based on RNA-Seq data from SRA, GTEx and TCGA. GeneFriends can perform single or multiple gene searches. The outputs are coexpressed gene lists which can be shown as GCNs. Additionally, biological term statistics are included and enrichment analysis is performed through DAVID.

**Correlation AnalyzeR** [184] performs *Homo sapiens* gene coexpression analysis based on reanalysed RNA-Seq read count data from ARCHS^4^. Available samples were re-processed and characterised based on tissue or cell-type. The webtool offers tissue-specific or global single gene, gene comparison and gene topology coexpression analyses. Single gene output is a coexpressed gene list along with a histogram depicting the frequency of correlation values of the coexpressed genes. A gene vs. gene scatterplot displays the correlation values of an input gene pair and the rest of the gene pool. In both cases, MSigDB-based [185] enrichment analysis results are also displayed below. A multiple gene coexpression analysis has similar output to the single gene search, highlighting only the correlations of the driver gene with the rest of the input genes. Finally, the gene list topology function clusters the input genes and subsequently performs the following analyses: PCA, variant gene heatmap creation and pathway enrichment analysis. Correlation AnalyzeR is available both as a webtool and a stand-alone R package.

**ImmuCo** [186] is the first web tool to perform coexpression analysis between any two genes in multiple immune cells in *Homo sapiens* or *Mus musculus*. ImmuCO outputs a scatter plot of the correlation values for each gene pair to illustrate the extent of correlation. A list of positively coexpressed genes can also be downloaded for each one of the query genes.

**Immuno-Navigator** [187] contains gene expression and coexpression data for immune system cells, from 4639 *Homo sapiens* and 3434 *Mus musculus* samples, covering 19 and 24 hematopoietic cell types, respectively. Immuno-Navigator was novel in addressing batch effect correction, thus improving the quality of both expression and correlation data. Immuno-Navigator offers a variety of coexpression results: coexpressed gene lists to a driver gene in different cell types, gene–gene correlation comparison scatterplots in all available samples, GCN construction with a gene list of interest as input, as well as multiple enrichment analyses.

**MyoMiner** [106] performs condition-specific coexpression analysis in *Homo sapiens* and *Mus musculus* normal and pathological muscle samples. Microarray data were collected from ArrayExpress and GEO and were meticulously quality controlled and batch-corrected. MyoMiner was the first webtool to perform microarray sample normalisation with SCAN and Brainarray custom CDF. On the website, samples can be filtered by organism (human or mouse) and cell-line strain, gender, age, anatomical part, or condition. By selecting a gene as a driver, the top coexpressed genes to the gene of interest are displayed, taking into account only the filtered samples. A GCN can be constructed by using the list of coexpressed genes as input. Finally, a comparison of the coexpression analyses of two different sample subsets can be performed.

### 7.3. Stand-Alone Gene Coexpression Applications

**Figure 9:**
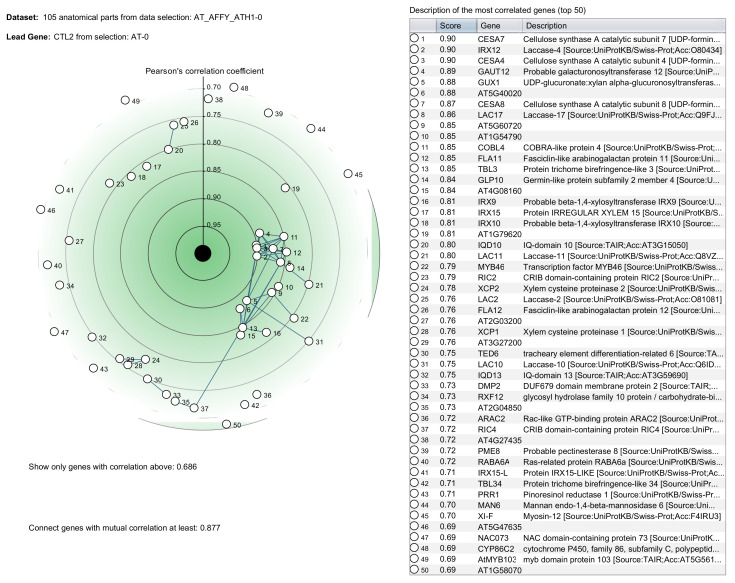
Output of positive coexpression analysis in Genevestigator with *CTL2* as driver gene. The “anatomy” sample dataset is used and the cut-offs of the inter-relationships of coexpressed genes are set to the default values.

**Genevestigator** [18] is a software tool for curated gene expression data. Genevestigator integrates thousands of microarray and RNA-Seq experiments (>320,000 datasets), offering a multitude of analyses such as differential expression, gene set enrichment and gene coexpression. In the latter, Genevestigator enables the user to choose the samples of interest through an internal search function and can discover positively or negatively coexpressed genes. Genevestigator not only outputs a coexpressed gene list to the driver gene but can also display possible coexpression interconnections of the resulting coexpressed genes (Figure 9). The resulting coexpressed gene list can be used as input for internal enrichment analysis.

**Weighted Gene Co-expression Network Analysis (WGCNA)** [130] is a widely used software package for weighted gene coexpression network construction implemented in R. A gene expression matrix of a set of samples, is required as input [188]. Initially, sample selection is performed as follows: hierarchical clustering of samples produces a dendrogram where samples are represented as leaves and leaves above a specified cut height and subclades with leaves less than a defined cutoff, are pruned. Using the expressions of the remaining samples, WGCNA constructs a coexpression network with weighted edges. To identify gene modules of the GCN, a TOM-based distance matrix is created from the GCN’s adjacency matrix, which is visualised as a dendrogram and subsequently separated into modules through dynamic tree cutting. Depending on the shape of the dendrogram, WGCNA’s parameters can be modified to produce an optimal number of modules [189]. Each gene module is represented as an eigengene. WGCNA may also visualise module-trait associations as a heatmap, by calculating PCCs between module eigengenes and quantitative traits. Values of such traits do not influence the constructed network of genes, but their addition can elucidate how each gene module influences each trait. Generally, WGCNA can be used in combination with other R packages which perform network analysis or functional biological term enrichment analysis of GCNs [139]. WGCNA facilitates finding hub genes of modules and the way modules associate with each other [190] and can predict the role of a gene of unknown function, based on the module it belongs to [188], as each module may be associated with certain biological pathways [139].

**QUalitative BIClustering 2 (QUBIC2)** [191] is a tool for performing biclustering in gene expression data. QUBIC2’s input is a gene expression matrix, which is converted into discrete values, using a left-truncated mixture gaussian (LTMG) model. After discretisation, the biclustering procedure is performed: A weighted graph is first constructed from the qualitative matrix, with the weights of the edges calculated as the amount of samples for which two genes have the same nonzero integer values. Then, through core biclustering identification and expansion, biclusters are produced. Finally, the biclusters are visualised as a heatmap and their statistical significance is evaluated through enrichment analysis. QUBIC2 has demonstrated robust results across data from microarrays and bulk and single-cell RNA-Seq.

**Factor Analysis for Bicluster Acquisition (FABIA)** [192] is an R-based bioconductor package performing biclustering. FABIA constitutes a multiplicative model with improved performance on heavy-tailed distribution data, as in the case of gene expressions. Depending on the input gene expression matrix, the appropriate Bayesian model is selected and applied. To discover the true biclusters, FABIA evaluates the produced biclusters by their information content. The tool’s output consists of a variety of plots for each bicluster, including heatmaps.

**Iterative Signature Algorithm (ISA)** [193] presents a characterisation of biclusters as transcription modules to be extracted from the expression data. A transcription module refers to a set of genes and a set of samples. A set of random gene and sample components is refined in an iterative procedure until it constitutes a transcription module, by applying a linear map without violating a threshold function. The algorithm outputs biclusters, while providing the ability to produce a heatmap. Subsequently, each bicluster can be plotted separately. Some biclusters may consist of overlapping genes and/or conditions [194].

**Figure 10:**
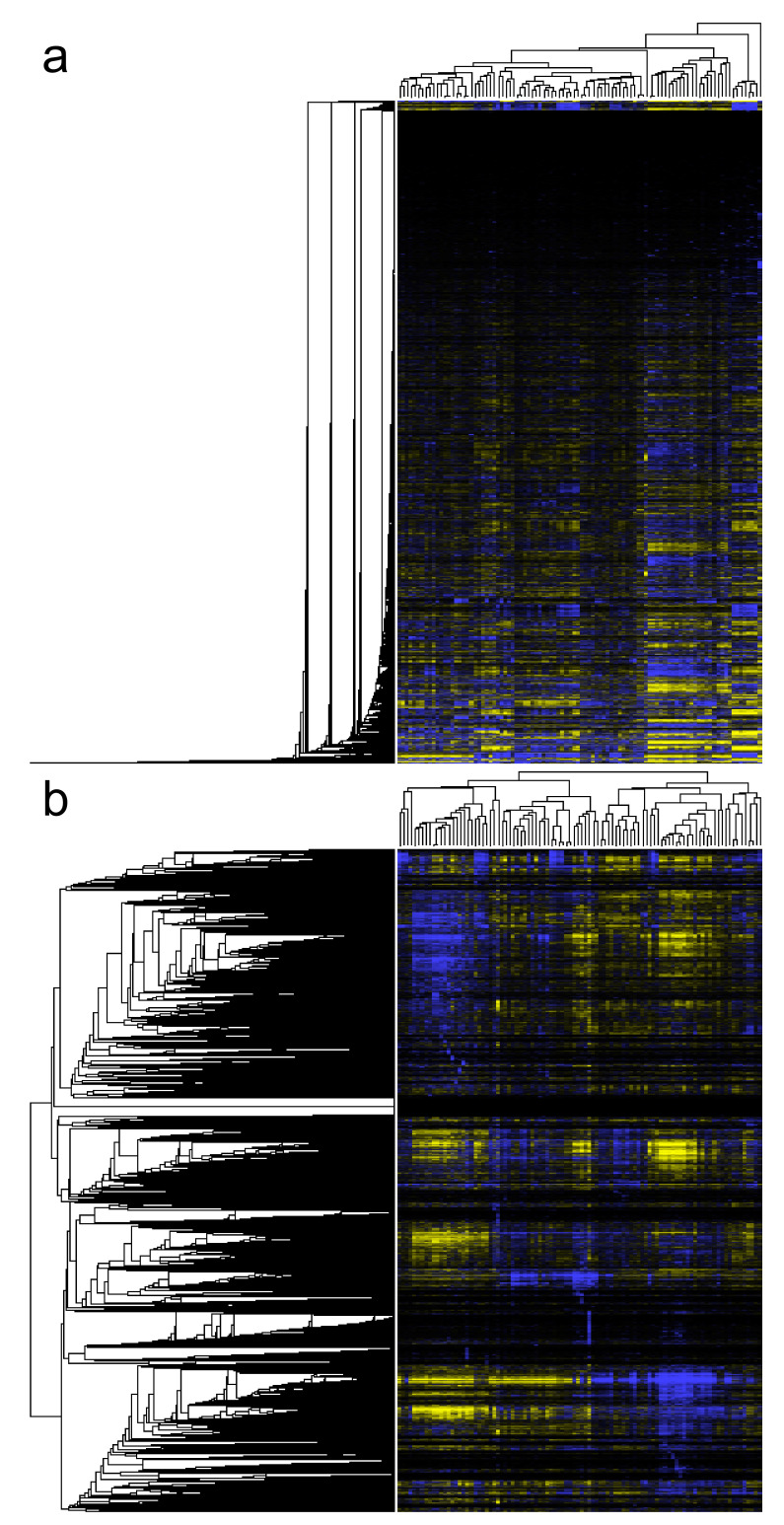
NCBI GEO Biclustering of samples and genes of GDS4562. Multiple biclusters of genes and samples of interest may be exported, plotted or linked to the corresponding entries of GEO Profiles. UPGMA clustering is performed using: (**a**) Euclidean distance; (**b**) Pearson correlation.

**NCBI GEO** [195] includes a visualisation tool for displaying cluster heat maps for each manually curated DataSet, essentially performing biclustering (Figure 10), using a variety of distance metrics (Euclidean distance, PCC or uncentred correlation coefficient) and clustering algorithms (single, complete or average linkage). To accelerate loading times, all clustering–distance metric combinations are pre-computed.

**CEMiTool** [196] is an R package for performing gene coexpression analysis and GCN construction. CEMiTool’s input is a gene expression matrix and performs the rest of the steps automatically: First, genes are filtered and using the remaining ones, a *β*-value is automatically selected for soft-thresholding. The resulting adjacency values are used to determine functional gene modules through DynamicTreeCut, as well as for the construction of a GCN. Users can optionally provide gene interaction data for additional interaction relationships in the resulting GCN. Various kinds of enrichment analysis can also be performed internally. Finally, the tool’s website version, webCEMiTool [197], has been successful in performing coexpression analysis using expression data derived from scRNA-Seq data.

**scLink** [198] is a pipeline for perfoming coexpression analysis on scRNA-Seq data, implemented in R. scLink uses a gene read count matrix deriving from scRNA-Seq as input and initially performs normalisation to produce gene expressions. Then, scImpute [101] is used to address the high amount of zero expressions. scLink uses a novel correlation measure which is based on an adaptation of the Gaussian graphical model in order to produce a coexpression matrix, which is ultimately used for the construction of a GCN. scLink has been successfully tested on mouse scRNA-Seq data.

## 8. Limitations and Perspectives in Coexpression Analysis

Gene coexpression analysis can be performed on the condition that an accurate estimation of gene expression is carried out, in a sufficient amount of samples of the same platform of a transcriptomic technology. Thus, coexpression analysis for non-model species may be limited by data availability.

A primary limitation of microarrays is that an organism can only be studied by using chips specifically designed for its own transcriptome. As microarray chip design is complicated and mass-production is costly, this technology is only available for a limited number of model species. Another intrinsic limitation of microarrays is their inability to produce expression values for genes for which there are no probes on the surface of the chip. Futhermore, probe cross-hybridisation may distort the estimation of the expression of genes of the same family and eventually their correlations with other genes.

Standard RNA-Seq pre-processing workflow requires a known genome and transcriptome, thus, the study of gene expression in non-model species, is possible as long as their genome and transcriptome are published. Gene expression analysis may also be performed in non-model species with no genome available if assembly and annotation of a de novo transcriptome, is performed prior to transcript count: based on RNA-Seq reads, de novo transcriptome assembly may be performed, e.g., using Trinity [199]. Then, the transcripts are characterised by matching their sequences to homologous genes of related organisms, and functionally annotated, using their corresponding biological terms, through an annotation pipeline, e.g., Trinotate [200]. Ultimately, transcript abundance is estimated by pseudoaligning RNA-Seq reads on the de novo transcriptome, using Salmon or Kallisto. These additional steps in the RNA-Seq workflow introduce extra assumptions which may reduce the overall quality of subsequent coexpression analysis and, to our knowledge, no coexpression tool based on it has yet been developed.

Bulk RNA-Seq estimates an “average” expression for each gene in the multitude of cells which comprises a biological sample. This might reduce the ability to detect the “fine-tuning” of corregulation of genes, a limitation which may be overcome using the singular high-resolution heterogeneous expression data derived from scRNA-Seq [201]. Any steps performed after the production of expression values in scRNA-Seq, like dimensionality reduction, should be ignored as they are irrelevant in coexpression analysis.

A strand-specific short-read sequencing technology that ensures one read per transcript, thus facilitating accurate gene expression estimation, is QuantSeq 3′ mRNA sequencing [202]. This technology can be efficiently integrated with current pipelines, such as Salmon [203]. As the probes of the most popular Affymetrix microarray chips target the 3′-end of the transcripts, the output of QuantSeq may be most comparable to that of microarrays, making it ideal for the comparison of microarray-based data of model organisms with RNA-Seq-based data of non-model organisms.

RNA-Seq feature count is normally performed at the gene level, thus ignoring differences in the expression level of alternative transcripts. Alternative transcripts may derive from alternative transcription start sites (TSSs), Transcription end sites (TESs) or splicing donor–splicing acceptor combinations. Isoforms of the same gene may possess different biological functions, e.g., TP73 isoforms whose coding sequence (CDS) includes a transactivation domain are considered apoptotic, while TP73 isoforms whose CDS lacks this transactivation domain, are considered anti-apoptotic [204]. Quantification of the expression of individual isoforms may be achieved by using methods for profiling the sequence of initiation of transcription [205] through STRIPE-seq [206] or Tn5Prime [207] or by using certain scRNA-Seq methods with increased read mapping across all transcripts and the ability to detect splice variants, such as Smart-seq2 [208]. Integrating the results of the aforementioned methods into coexpression analysis would facilitate the study of the specific functions of protein isoforms and their coexpressed partners. Nevertheless, most biological terms are assigned to genes and are not isoform-specific. A notable exception is the transcription factors that control each alternative promoter. Isoform-specific biological term enrichments could provide more insight on the coexpression landscape.

The most commonly used measures for deducing gene similarity are Euclidean distance and Pearson and Spearman correlation coefficients and there are many R packages which can be used to calculate them. While Euclidean distance is sensitive to scaling and differences in average expression levels [137], resulting in imbalanced correlation trees with a characteristic “ladder” effect (Figure 10a), correlation coefficients result in more balanced trees (Figure 10b). Pearson correlation is mostly preferred to Spearman, showing slightly better results in coexpression network creation (Spearman only performs better in small sample number datasets) [97]. Most of the other measures, such as TOM similarity or LS are intricate transformations of PCC. However, correlation coefficients may not be effective in studying gene coexpression based on scRNA-Seq data, due to the high heterogeneity and noise in scRNA-Seq expression values [198]. Thus, new correlation methods may need to be invented and extensively tested, before they become mainstream.

## 9. General Guidelines for Coexpression Tool Selection

Coexpression tools are used to produce verifiable biological hypotheses, through which the users can create their experiments for the identification of gene partners or novel gene functions. A simpler use is the provision of an additional line of evidence in an already completed experimental analysis. Although gene coexpression tools produce comparable results, there are notable discrepancies among them, since they are based on different transcriptomic data and coexpression analysis workflows.

At first, the user should decide whether the tool for the species of interest should be global or tissue/cell-type specific. Then, a collection of global or tissue-specific tools, depending on the previous selection, might be run for analysis and the user could form a consensus list of coexpressed genes that are present in the results of the majority of the tools. Alternatively, the user might assess the performance of each tool, based on various indications for an efficient depiction of the coexpression landscape. First of all, the number of samples used by each tool is an important factor, with higher sample numbers resulting in more reliable coexpression relationships, as a small sample number might introduce sparse correlations [3]. Sample variability is equally important to ensure that the dataset is not skewed towards a certain tissue, when global coexpression is studied. In addition, high-quality samples and the application of batch correction increases the quality of coexpression [97,122,209,210,211].

Tools that are based on up-to-date genome/transcriptome data or biological terms are preferable, e.g., microarray-based tools using a custom CDF are innately better than those using the default one. The mathematical rigor of the underlying statistics of a coexpression tool may also improve its performance. This might be assessed by the complexity of the correlation calculation method, as well as by the resulting depiction of coexpression. The latter can be evaluated by the ability of the tool to reproduce known biology: The output of each tool could be cross-checked with the existing bibliography by searching for validated gene partners in the coexpression lists or validated biological processes in the statistically significant enriched biological terms. Enrichment analysis can be performed either internally, by some coexpression tools, or by exporting the coexpressed gene list to external webtools such as WebGestalt, where either pre-set or user-defined reference gene lists may also be used.

## Data Availability

Not applicable.

## Supplementary Materials

The following supporting information can be downloaded at: https://www.mdpi.com/article/10.3390/biology11071019/s1, Table S1: Detailed information on available coexpression webtools.

## References

[1] Schneider M.V., Orchard S., Mayer B. (2011). Omics Technologies, Data and Bioinformatics Principles. Bioinformatics for Omics Data: Methods and Protocols.

[2] Barabasi A.L., Oltvai Z.N. (2004). Network biology: Understanding the cell’s functional organization. Nat. Rev. Genet..

[3] Usadel B., Obayashi T., Mutwil M., Giorgi F.M., Bassel G.W., Tanimoto M., Chow A., Steinhauser D., Persson S., Provart N.J. (2009). Co-expression tools for plant biology: Opportunities for hypothesis generation and caveats. Plant Cell Environ..

[4] Emamjomeh A., Saboori Robat E., Zahiri J., Solouki M., Khosravi P. (2017). Gene co-expression network reconstruction: A review on computational methods for inferring functional information from plant-based expression data. Plant Biotechnol. Rep..

[5] Pavlopoulos G.A., Secrier M., Moschopoulos C.N., Soldatos T.G., Kossida S., Aerts J., Schneider R., Bagos P.G. (2011). Using graph theory to analyze biological networks. BioData Min..

[6] Pellegrini M., Haynor D., Johnson J.M. (2004). Protein interaction networks. Expert Rev. Proteom..

[7] Emmert-Streib F., Dehmer M., Haibe-Kains B. (2014). Gene regulatory networks and their applications: Understanding biological and medical problems in terms of networks. Front. Cell Dev. Biol..

[8] Albert R., DasGupta B., Dondi R., Kachalo S., Sontag E., Zelikovsky A., Westbrooks K. (2007). A novel method for signal transduction network inference from indirect experimental evidence. J. Comput. Biol..

[9] Jeong H., Tombor B., Albert R., Oltvai Z.N., Barabasi A.L. (2000). The large-scale organization of metabolic networks. Nature.

[10] Tieri P., Farina L., Petti M., Astolfi L., Paci P., Castiglione F., Ranganathan S., Gribskov M., Nakai K., Schönbach C. (2019). Network Inference and Reconstruction in Bioinformatics. Encyclopedia of Bioinformatics and Computational Biology.

[11] Fionda V., Ranganathan S., Gribskov M., Nakai K., Schönbach C. (2019). Networks in Biology. Encyclopedia of Bioinformatics and Computational Biology.

[12] Serin E.A.R., Nijveen H., Hilhorst H.W.M., Ligterink W. (2016). Learning from Co-expression Networks: Possibilities and Challenges. Front. Plant Sci..

[13] Michalopoulos I., Pavlopoulos G.A., Malatras A., Karelas A., Kostadima M.A., Schneider R., Kossida S. (2012). Human gene correlation analysis (HGCA): A tool for the identification of transcriptionally co-expressed genes. BMC Res. Notes.

[14] Petereit J., Smith S., Harris F.C., Schlauch K.A. (2016). Petal: Co-expression network modelling in R. BMC Syst. Biol..

[15] He F., Maslov S. (2016). Pan- and core- network analysis of co-expression genes in a model plant. Sci. Rep..

[16] Liseron-Monfils C., Ware D. (2015). Revealing gene regulation and associations through biological networks. Curr. Plant Biol..

[17] Obayashi T., Kagaya Y., Aoki Y., Tadaka S., Kinoshita K. (2019). COXPRESdb v7: A gene coexpression database for 11 animal species supported by 23 coexpression platforms for technical evaluation and evolutionary inference. Nucleic Acids Res..

[18] Hruz T., Laule O., Szabo G., Wessendorp F., Bleuler S., Oertle L., Widmayer P., Gruissem W., Zimmermann P. (2008). Genevestigator v3: A reference expression database for the meta-analysis of transcriptomes. Adv. Bioinform..

[19] Jupiter D., Chen H., VanBuren V. (2009). STARNET 2: A web-based tool for accelerating discovery of gene regulatory networks using microarray co-expression data. BMC Bioinform..

[20] Yang S., Kim C.Y., Hwang S., Kim E., Kim H., Shim H., Lee I. (2017). COEXPEDIA: Exploring biomedical hypotheses via co-expressions associated with medical subject headings (MeSH). Nucleic Acids Res..

[21] Lachmann A., Torre D., Keenan A.B., Jagodnik K.M., Lee H.J., Wang L., Silverstein M.C., Ma’ayan A. (2018). Massive mining of publicly available RNA-seq data from human and mouse. Nat. Commun..

[22] Obayashi T., Aoki Y., Tadaka S., Kagaya Y., Kinoshita K. (2018). ATTED-II in 2018: A Plant Coexpression Database Based on Investigation of the Statistical Property of the Mutual Rank Index. Plant Cell Physiol..

[23] Zogopoulos V.L., Saxami G., Malatras A., Angelopoulou A., Jen C.H., Duddy W.J., Daras G., Hatzopoulos P., Westhead D.R., Michalopoulos I. (2021). Arabidopsis Coexpression Tool: A tool for gene coexpression analysis in *Arabidopsis thaliana*. iScience.

[24] Leal L.G., Lopez C., Lopez-Kleine L. (2014). Construction and comparison of gene co-expression networks shows complex plant immune responses. PeerJ.

[25] Narise T., Sakurai N., Obayashi T., Ohta H., Shibata D. (2017). Co-expressed Pathways DataBase for Tomato: A database to predict pathways relevant to a query gene. BMC Genom..

[26] Kawahara Y., Oono Y., Wakimoto H., Ogata J., Kanamori H., Sasaki H., Mori S., Matsumoto T., Itoh T. (2016). TENOR: Database for Comprehensive mRNA-Seq Experiments in Rice. Plant Cell Physiol..

[27] Xia L., Zou D., Sang J., Xu X., Yin H., Li M., Wu S., Hu S., Hao L., Zhang Z. (2017). Rice Expression Database (RED): An integrated RNA-Seq-derived gene expression database for rice. J. Genet. Genom..

[28] Yim W.C., Yu Y., Song K., Jang C.S., Lee B.M. (2013). PLANEX: The plant co-expression database. BMC Plant Biol..

[29] Proost S., Mutwil M. (2017). PlaNet: Comparative Co-Expression Network Analyses for Plants. Methods Mol. Biol..

[30] Van Dam S., Craig T., de Magalhaes J.P. (2015). GeneFriends: A human RNA-seq-based gene and transcript co-expression database. Nucleic Acids Res..

[31] Franz M., Rodriguez H., Lopes C., Zuberi K., Montojo J., Bader G.D., Morris Q. (2018). GeneMANIA update 2018. Nucleic Acids Res..

[32] van Dam S., Vosa U., van der Graaf A., Franke L., de Magalhaes J.P. (2018). Gene co-expression analysis for functional classification and gene-disease predictions. Brief. Bioinform..

[33] Peng J., Wang T., Huc J., Wang Y., Chen J. (2016). Constructing Networks of Organelle Functional Modules in Arabidopsis. Curr. Genom..

[34] Schena M., Shalon D., Davis R.W., Brown P.O. (1995). Quantitative monitoring of gene expression patterns with a complementary DNA microarray. Science.

[35] Wang Z., Gerstein M., Snyder M. (2009). RNA-Seq: A revolutionary tool for transcriptomics. Nat. Rev. Genet..

[36] Barrett T., Wilhite S.E., Ledoux P., Evangelista C., Kim I.F., Tomashevsky M., Marshall K.A., Phillippy K.H., Sherman P.M., Holko M. (2013). NCBI GEO: Archive for functional genomics data sets—Update. Nucleic Acids Res..

[37] Parkinson H., Kapushesky M., Shojatalab M., Abeygunawardena N., Coulson R., Farne A., Holloway E., Kolesnykov N., Lilja P., Lukk M. (2007). ArrayExpress–A public database of microarray experiments and gene expression profiles. Nucleic Acids Res..

[38] Papatheodorou I., Moreno P., Manning J., Fuentes A.M., George N., Fexova S., Fonseca N.A., Fullgrabe A., Green M., Huang N. (2020). Expression Atlas update: From tissues to single cells. Nucleic Acids Res..

[39] Kodama Y., Shumway M., Leinonen R., International Nucleotide Sequence Database C. (2012). The Sequence Read Archive: Explosive growth of sequencing data. Nucleic Acids Res..

[40] GTEx Consortium (2013). The Genotype-Tissue Expression (GTEx) project. Nat. Genet..

[41] Hutter C., Zenklusen J.C. (2018). The Cancer Genome Atlas: Creating Lasting Value beyond Its Data. Cell.

[42] Amid C., Alako B.T.F., Balavenkataraman Kadhirvelu V., Burdett T., Burgin J., Fan J., Harrison P.W., Holt S., Hussein A., Ivanov E. (2020). The European Nucleotide Archive in 2019. Nucleic Acids Res..

[43] Aoki K., Ogata Y., Shibata D. (2007). Approaches for extracting practical information from gene co-expression networks in plant biology. Plant Cell Physiol..

[44] Langfelder P., Horvath S. WGCNA Package FAQ. https://horvath.genetics.ucla.edu/html/CoexpressionNetwork/Rpackages/WGCNA/faq.html.

[45] Lockhart D.J., Dong H., Byrne M.C., Follettie M.T., Gallo M.V., Chee M.S., Mittmann M., Wang C., Kobayashi M., Horton H. (1996). Expression monitoring by hybridization to high-density oligonucleotide arrays. Nat. Biotechnol..

[46] Wolber P.K., Collins P.J., Lucas A.B., De Witte A., Shannon K.W. (2006). The Agilent in situ-synthesized microarray platform. Methods Enzymol..

[47] Kuhn K., Baker S.C., Chudin E., Lieu M.H., Oeser S., Bennett H., Rigault P., Barker D., McDaniel T.K., Chee M.S. (2004). A novel, high-performance random array platform for quantitative gene expression profiling. Genome Res..

[48] Hubbell E., Liu W.M., Mei R. (2002). Robust estimators for expression analysis. Bioinformatics.

[49] Irizarry R.A., Bolstad B.M., Collin F., Cope L.M., Hobbs B., Speed T.P. (2003). Summaries of Affymetrix GeneChip probe level data. Nucleic Acids Res..

[50] Wu Z., Irizarry R.A., Gentleman R., Martinez-Murillo F., Spencer F. (2004). A Model-Based Background Adjustment for Oligonucleotide Expression Arrays. J. Am. Stat. Assoc..

[51] Hubbell E. Affymetrix Technical Notes: Guide to Probe Logarithmic Intensity Error (PLIER) Estimation. http://tools.thermofisher.com/content/sfs/brochures/plier_technote.pdf.

[52] Piccolo S.R., Sun Y., Campbell J.D., Lenburg M.E., Bild A.H., Johnson W.E. (2012). A single-sample microarray normalization method to facilitate personalized-medicine workflows. Genomics.

[53] Zogopoulos V.L., Malatras A., Michalopoulos I. (2022). Gene coexpression analysis in Arabidopsis thaliana based on public microarray data. STAR Protoc..

[54] R Core Team R: A Language and Environment for Statistical Computing. https://cran.r-project.org/doc/manuals/r-release/fullrefman.pdf.

[55] Eijssen L.M., Jaillard M., Adriaens M.E., Gaj S., de Groot P.J., Muller M., Evelo C.T. (2013). User-friendly solutions for microarray quality control and pre-processing on ArrayAnalysis.org. Nucleic Acids Res..

[56] Applied Biosystems Applied Biosystems 3730 and 3730xl DNA Analyzers. http://tools.thermofisher.com/content/sfs/brochures/cms_042636.pdf.

[57] Jain M., Olsen H.E., Paten B., Akeson M. (2016). The Oxford Nanopore MinION: Delivery of nanopore sequencing to the genomics community. Genome Biol..

[58] Bentley D.R., Balasubramanian S., Swerdlow H.P., Smith G.P., Milton J., Brown C.G., Hall K.P., Evers D.J., Barnes C.L., Bignell H.R. (2008). Accurate whole human genome sequencing using reversible terminator chemistry. Nature.

[59] Margulies M., Egholm M., Altman W.E., Attiya S., Bader J.S., Bemben L.A., Berka J., Braverman M.S., Chen Y.J., Chen Z. (2005). Genome sequencing in microfabricated high-density picolitre reactors. Nature.

[60] Schadt E.E., Turner S., Kasarskis A. (2010). A window into third-generation sequencing. Hum. Mol. Genet..

[61] Branton D., Deamer D.W., Marziali A., Bayley H., Benner S.A., Butler T., Di Ventra M., Garaj S., Hibbs A., Huang X. (2008). The potential and challenges of nanopore sequencing. Nat. Biotechnol..

[62] Cock P.J., Fields C.J., Goto N., Heuer M.L., Rice P.M. (2010). The Sanger FASTQ file format for sequences with quality scores, and the Solexa/Illumina FASTQ variants. Nucleic Acids Res..

[63] Hong M., Tao S., Zhang L., Diao L.T., Huang X., Huang S., Xie S.J., Xiao Z.D., Zhang H. (2020). RNA sequencing: New technologies and applications in cancer research. J. Hematol. Oncol..

[64] Macmanes M.D. (2014). On the optimal trimming of high-throughput mRNA sequence data. Front. Genet..

[65] Andrews S. FastQC: A Quality Control Tool for High Throughput Sequence Data. https://www.bioinformatics.babraham.ac.uk/projects/fastqc/.

[66] Ewels P., Magnusson M., Lundin S., Kaller M. (2016). MultiQC: Summarize analysis results for multiple tools and samples in a single report. Bioinformatics.

[67] Fukasawa Y., Ermini L., Wang H., Carty K., Cheung M.S. (2020). LongQC: A Quality Control Tool for Third Generation Sequencing Long Read Data. G3 Genes Genomes Genet..

[68] Martin M. (2011). Cutadapt removes adapter sequences from high-throughput sequencing reads. EMBnet. J..

[69] Chen S., Zhou Y., Chen Y., Gu J. (2018). Fastp: An ultra-fast all-in-one FASTQ preprocessor. Bioinformatics.

[70] Bolger A.M., Lohse M., Usadel B. (2014). Trimmomatic: A flexible trimmer for Illumina sequence data. Bioinformatics.

[71] Kim D., Pertea G., Trapnell C., Pimentel H., Kelley R., Salzberg S.L. (2013). TopHat2: Accurate alignment of transcriptomes in the presence of insertions, deletions and gene fusions. Genome Biol..

[72] Kim D., Paggi J.M., Park C., Bennett C., Salzberg S.L. (2019). Graph-based genome alignment and genotyping with HISAT2 and HISAT-genotype. Nat. Biotechnol..

[73] Boratyn G.M., Thierry-Mieg J., Thierry-Mieg D., Busby B., Madden T.L. (2019). Magic-BLAST, an accurate RNA-seq aligner for long and short reads. BMC Bioinform..

[74] Marić J., Sović I., Križanović K., Nagarajan N., Šikić M. (2019). Graphmap2—Splice-aware RNA-seq mapper for long reads. bioRxiv.

[75] Lin H.N., Hsu W.L. (2018). DART: A fast and accurate RNA-seq mapper with a partitioning strategy. Bioinformatics.

[76] Liu B., Liu Y., Li J., Guo H., Zang T., Wang Y. (2019). deSALT: Fast and accurate long transcriptomic read alignment with de Bruijn graph-based index. Genome Biol..

[77] Langmead B., Salzberg S.L. (2012). Fast gapped-read alignment with Bowtie 2. Nat. Methods.

[78] Li H. (2018). Minimap2: Pairwise alignment for nucleotide sequences. Bioinformatics.

[79] Dobin A., Davis C.A., Schlesinger F., Drenkow J., Zaleski C., Jha S., Batut P., Chaisson M., Gingeras T.R. (2013). STAR: Ultrafast universal RNA-seq aligner. Bioinformatics.

[80] Wu T.D., Reeder J., Lawrence M., Becker G., Brauer M.J. (2016). GMAP and GSNAP for Genomic Sequence Alignment: Enhancements to Speed, Accuracy, and Functionality. Methods Mol. Biol..

[81] Li H. (2013). Aligning sequence reads, clone sequences and assembly contigs with BWA-MEM. arXiv.

[82] Li H., Handsaker B., Wysoker A., Fennell T., Ruan J., Homer N., Marth G., Abecasis G., Durbin R., Genome Project Data Processing S. (2009). The Sequence Alignment/Map format and SAMtools. Bioinformatics.

[83] Stein L. Generic Feature Format Version 3 (GFF3). https://github.com/The-Sequence-Ontology/Specifications/blob/master/gff3.md.

[84] Trapnell C., Williams B.A., Pertea G., Mortazavi A., Kwan G., van Baren M.J., Salzberg S.L., Wold B.J., Pachter L. (2010). Transcript assembly and quantification by RNA-Seq reveals unannotated transcripts and isoform switching during cell differentiation. Nat. Biotechnol..

[85] Liao Y., Smyth G.K., Shi W. (2014). featureCounts: An efficient general purpose program for assigning sequence reads to genomic features. Bioinformatics.

[86] Anders S., Pyl P.T., Huber W. (2015). HTSeq—A Python framework to work with high-throughput sequencing data. Bioinformatics.

[87] Dillies M.A., Rau A., Aubert J., Hennequet-Antier C., Jeanmougin M., Servant N., Keime C., Marot G., Castel D., Estelle J. (2013). A comprehensive evaluation of normalization methods for Illumina high-throughput RNA sequencing data analysis. Brief. Bioinform..

[88] Bolstad B.M., Irizarry R.A., Astrand M., Speed T.P. (2003). A comparison of normalization methods for high density oligonucleotide array data based on variance and bias. Bioinformatics.

[89] Bullard J.H., Purdom E., Hansen K.D., Dudoit S. (2010). Evaluation of statistical methods for normalization and differential expression in mRNA-Seq experiments. BMC Bioinform..

[90] Wagner G.P., Kin K., Lynch V.J. (2012). Measurement of mRNA abundance using RNA-seq data: RPKM measure is inconsistent among samples. Theory Biosci..

[91] Mortazavi A., Williams B.A., McCue K., Schaeffer L., Wold B. (2008). Mapping and quantifying mammalian transcriptomes by RNA-Seq. Nat. Methods.

[92] Robinson M.D., Oshlack A. (2010). A scaling normalization method for differential expression analysis of RNA-seq data. Genome Biol..

[93] Love M.I., Huber W., Anders S. (2014). Moderated estimation of fold change and dispersion for RNA-seq data with DESeq2. Genome Biol..

[94] Hicks S.C., Okrah K., Paulson J.N., Quackenbush J., Irizarry R.A., Bravo H.C. (2018). Smooth quantile normalization. Biostatistics.

[95] Bray N.L., Pimentel H., Melsted P., Pachter L. (2016). Near-optimal probabilistic RNA-seq quantification. Nat. Biotechnol..

[96] Patro R., Duggal G., Love M.I., Irizarry R.A., Kingsford C. (2017). Salmon provides fast and bias-aware quantification of transcript expression. Nat. Methods.

[97] Vandenbon A. (2022). Evaluation of critical data processing steps for reliable prediction of gene co-expression from large collections of RNA-seq data. PLoS ONE.

[98] Tang F., Barbacioru C., Wang Y., Nordman E., Lee C., Xu N., Wang X., Bodeau J., Tuch B.B., Siddiqui A. (2009). mRNA-Seq whole-transcriptome analysis of a single cell. Nat. Methods.

[99] Hwang B., Lee J.H., Bang D. (2018). Single-cell RNA sequencing technologies and bioinformatics pipelines. Exp. Mol. Med..

[100] Chen G., Ning B., Shi T. (2019). Single-Cell RNA-Seq Technologies and Related Computational Data Analysis. Front. Genet..

[101] Li W.V., Li J.J. (2018). An accurate and robust imputation method scImpute for single-cell RNA-seq data. Nat. Commun..

[102] Huang M., Wang J., Torre E., Dueck H., Shaffer S., Bonasio R., Murray J.I., Raj A., Li M., Zhang N.R. (2018). SAVER: Gene expression recovery for single-cell RNA sequencing. Nat. Methods.

[103] Van Dijk D., Sharma R., Nainys J., Yim K., Kathail P., Carr A.J., Burdziak C., Moon K.R., Chaffer C.L., Pattabiraman D. (2018). Recovering Gene Interactions from Single-Cell Data Using Data Diffusion. Cell.

[104] Dai M., Wang P., Boyd A.D., Kostov G., Athey B., Jones E.G., Bunney W.E., Myers R.M., Speed T.P., Akil H. (2005). Evolving gene/transcript definitions significantly alter the interpretation of GeneChip data. Nucleic Acids Res..

[105] Chen L., Sun F., Yang X., Jin Y., Shi M., Wang L., Shi Y., Zhan C., Wang Q. (2017). Correlation between RNA-Seq and microarrays results using TCGA data. Gene.

[106] Malatras A., Michalopoulos I., Duguez S., Butler-Browne G., Spuler S., Duddy W.J. (2020). MyoMiner: Explore gene co-expression in normal and pathological muscle. BMC Med. Genom..

[107] Leek J.T., Scharpf R.B., Bravo H.C., Simcha D., Langmead B., Johnson W.E., Geman D., Baggerly K., Irizarry R.A. (2010). Tackling the widespread and critical impact of batch effects in high-throughput data. Nat. Rev. Genet..

[108] Pearson K. (1901). LIII. On lines and planes of closest fit to systems of points in space. Lond. Edinb. Dublin Philos. Mag..

[109] Sokal R.R., Michener C.D. (1958). A statistical method for evaluating systematic relationships. Univ. Kansas Sci. Bull..

[110] Johnson W.E., Li C., Rabinovic A. (2007). Adjusting batch effects in microarray expression data using empirical Bayes methods. Biostatistics.

[111] Leek J.T., Storey J.D. (2008). A general framework for multiple testing dependence. Proc. Natl. Acad. Sci. USA.

[112] Buettner F., Pratanwanich N., McCarthy D.J., Marioni J.C., Stegle O. (2017). f-scLVM: Scalable and versatile factor analysis for single-cell RNA-seq. Genome Biol..

[113] Haghverdi L., Lun A.T.L., Morgan M.D., Marioni J.C. (2018). Batch effects in single-cell RNA-sequencing data are corrected by matching mutual nearest neighbors. Nat. Biotechnol..

[114] Buttner M., Miao Z., Wolf F.A., Teichmann S.A., Theis F.J. (2019). A test metric for assessing single-cell RNA-seq batch correction. Nat. Methods.

[115] Minkowski H. (1910). Geometrie Der Zahlen.

[116] Pearson K. (1895). VII. Note on regression and inheritance in the case of two parents. Proc. R. Soc. Lond..

[117] Amaratunga D., Cabrera J. (2001). Analysis of Data From Viral DNA Microchips. J. Am. Stat. Assoc..

[118] Jaskowiak P.A., Campello R.J., Costa I.G. (2014). On the selection of appropriate distances for gene expression data clustering. BMC Bioinform..

[119] Spearman C. (1904). ‘General intelligence’, objectively determined and measured. Am. J. Psychol..

[120] Myers J.L., Well A.D. (2003). Research Design and Statistical Analysis.

[121] Kendall M.G. (1938). A new measure of rank correlation. Biometrika.

[122] Kumari S., Nie J., Chen H.S., Ma H., Stewart R., Li X., Lu M.Z., Taylor W.M., Wei H. (2012). Evaluation of gene association methods for coexpression network construction and biological knowledge discovery. PLoS ONE.

[123] Obayashi T., Hayashi S., Saeki M., Ohta H., Kinoshita K. (2009). ATTED-II provides coexpressed gene networks for Arabidopsis. Nucleic Acids Res..

[124] Obayashi T., Hibara H., Kagaya Y., Aoki Y., Kinoshita K. (2022). ATTED-II v11: A Plant Gene Coexpression Database Using a Sample Balancing Technique by Subagging of Principal Components. Plant Cell Physiol..

[125] Bansal M., Belcastro V., Ambesi-Impiombato A., di Bernardo D. (2007). How to infer gene networks from expression profiles. Mol. Syst. Biol..

[126] Shannon C.E. (1948). A Mathematical Theory of Communication. Bell Syst. Tech. J..

[127] Steuer R., Kurths J., Daub C.O., Weise J., Selbig J. (2002). The mutual information: Detecting and evaluating dependencies between variables. Bioinformatics.

[128] Mousavi A., Baraniuk R.G. An information-theoretic measure of dependency among variables in large datasets. Proceedings of the 2015 53rd Annual Allerton Conference on Communication Control, and Computing (Allerton).

[129] Obayashi T., Kinoshita K. (2010). Coexpression landscape in ATTED-II: Usage of gene list and gene network for various types of pathways. J. Plant Res..

[130] Zhang B., Horvath S. (2005). A general framework for weighted gene co-expression network analysis. Stat. Appl. Genet. Mol. Biol..

[131] Borate B.R., Chesler E.J., Langston M.A., Saxton A.M., Voy B.H. (2009). Comparison of threshold selection methods for microarray gene co-expression matrices. BMC Res. Notes.

[132] Ala U., Piro R.M., Grassi E., Damasco C., Silengo L., Oti M., Provero P., Di Cunto F. (2008). Prediction of human disease genes by human-mouse conserved coexpression analysis. PLoS Comput. Biol..

[133] De la Fuente A. (2010). From ‘differential expression’ to ‘differential networking’—Identification of dysfunctional regulatory networks in diseases. Trends Genet..

[134] Christensen C., Thakar J., Albert R. (2007). Systems-level insights into cellular regulation: Inferring, analysing, and modelling intracellular networks. IET Syst. Biol..

[135] Shannon P., Markiel A., Ozier O., Baliga N.S., Wang J.T., Ramage D., Amin N., Schwikowski B., Ideker T. (2003). Cytoscape: A software environment for integrated models of biomolecular interaction networks. Genome Res..

[136] Franz M., Lopes C.T., Huck G., Dong Y., Sumer O., Bader G.D. (2016). Cytoscape.js: A graph theory library for visualisation and analysis. Bioinformatics.

[137] D’Haeseleer P. (2005). How does gene expression clustering work?. Nat. Biotechnol..

[138] Perkins A.D., Langston M.A. (2009). Threshold selection in gene co-expression networks using spectral graph theory techniques. BMC Bioinform..

[139] Langfelder P., Horvath S. (2008). WGCNA: An R package for weighted correlation network analysis. BMC Bioinform..

[140] Jen C.H., Manfield I.W., Michalopoulos I., Pinney J.W., Willats W.G., Gilmartin P.M., Westhead D.R. (2006). The Arabidopsis co-expression tool (ACT): A WWW-based tool and database for microarray-based gene expression analysis. Plant J..

[141] Manfield I.W., Jen C.H., Pinney J.W., Michalopoulos I., Bradford J.R., Gilmartin P.M., Westhead D.R. (2006). Arabidopsis Co-expression Tool (ACT): Web server tools for microarray-based gene expression analysis. Nucleic Acids Res..

[142] Chen P., Wang F., Feng J., Zhou R., Chang Y., Liu J., Zhao Q. (2017). Co-expression network analysis identified six hub genes in association with metastasis risk and prognosis in hepatocellular carcinoma. Oncotarget.

[143] Yuan L., Chen L., Qian K., Qian G., Wu C.L., Wang X., Xiao Y. (2017). Co-expression network analysis identified six hub genes in association with progression and prognosis in human clear cell renal cell carcinoma (ccRCC). Genom. Data.

[144] Olsen G. The ”Newick’s 8:45” Tree Format Standard. https://evolution.genetics.washington.edu/phylip/newick_doc.html.

[145] Hartigan J.A. (1972). Direct Clustering of a Data Matrix. J. Am. Stat. Assoc..

[146] Padilha V.A., Campello R.J.G.B. (2017). A systematic comparative evaluation of biclustering techniques. BMC Bioinform..

[147] Eren K., Deveci M., Kucuktunc O., Catalyurek U.V. (2012). A comparative analysis of biclustering algorithms for gene expression data. Brief. Bioinform..

[148] Hartigan J. (1975). Clustering Algorithms.

[149] Heyer L.J., Kruglyak S., Yooseph S. (1999). Exploring expression data: Identification and analysis of coexpressed genes. Genome Res..

[150] Tamayo P., Slonim D., Mesirov J., Zhu Q., Kitareewan S., Dmitrovsky E., Lander E.S., Golub T.R. (1999). Interpreting patterns of gene expression with self-organizing maps: Methods and application to hematopoietic differentiation. Proc. Natl. Acad. Sci. USA.

[151] Farris J.S. (1969). On the Cophenetic Correlation Coefficient. Syst. Biol..

[152] Saraçli S., Doğan N., Doğan İ. (2013). Comparison of hierarchical cluster analysis methods by cophenetic correlation. J. Inequal. Appl..

[153] Fisher R.A. (1922). On the Interpretation of χ2 from Contingency Tables, and the Calculation of P. J. R. Stat. Soc..

[154] Subramanian A., Tamayo P., Mootha V.K., Mukherjee S., Ebert B.L., Gillette M.A., Paulovich A., Pomeroy S.L., Golub T.R., Lander E.S. (2005). Gene set enrichment analysis: A knowledge-based approach for interpreting genome-wide expression profiles. Proc. Natl. Acad. Sci. USA.

[155] Benjamini Y., Hochberg Y. (1995). Controlling the false discovery rate: A practical and powerful approach to multiple testing. J. Royal Stat. Soc. Ser. B.

[156] Gene Ontology Consortium (2021). The Gene Ontology resource: Enriching a GOld mine. Nucleic Acids Res..

[157] Kanehisa M., Furumichi M., Tanabe M., Sato Y., Morishima K. (2017). KEGG: New perspectives on genomes, pathways, diseases and drugs. Nucleic Acids Res..

[158] Mistry J., Chuguransky S., Williams L., Qureshi M., Salazar G.A., Sonnhammer E.L.L., Tosatto S.C.E., Paladin L., Raj S., Richardson L.J. (2021). Pfam: The protein families database in 2021. Nucleic Acids Res..

[159] Pinero J., Ramirez-Anguita J.M., Sauch-Pitarch J., Ronzano F., Centeno E., Sanz F., Furlong L.I. (2020). The DisGeNET knowledge platform for disease genomics: 2019 update. Nucleic Acids Res..

[160] Castro-Mondragon J.A., Riudavets-Puig R., Rauluseviciute I., Berhanu Lemma R., Turchi L., Blanc-Mathieu R., Lucas J., Boddie P., Khan A., Manosalva Perez N. (2022). JASPAR 2022: The 9th release of the open-access database of transcription factor binding profiles. Nucleic Acids Res..

[161] Encode Project Consortium (2020). Expanded encyclopaedias of DNA elements in the human and mouse genomes. Nature.

[162] Raudvere U., Kolberg L., Kuzmin I., Arak T., Adler P., Peterson H., Vilo J. (2019). g:Profiler: A web server for functional enrichment analysis and conversions of gene lists (2019 update). Nucleic Acids Res..

[163] Kuleshov M.V., Jones M.R., Rouillard A.D., Fernandez N.F., Duan Q., Wang Z., Koplev S., Jenkins S.L., Jagodnik K.M., Lachmann A. (2016). Enrichr: A comprehensive gene set enrichment analysis web server 2016 update. Nucleic Acids Res..

[164] Liao Y., Wang J., Jaehnig E.J., Shi Z., Zhang B. (2019). WebGestalt 2019: Gene set analysis toolkit with revamped UIs and APIs. Nucleic Acids Res..

[165] Thanati F., Karatzas E., Baltoumas F.A., Stravopodis D.J., Eliopoulos A.G., Pavlopoulos G.A. (2021). FLAME: A Web Tool for Functional and Literature Enrichment Analysis of Multiple Gene Lists. Biology.

[166] Huang D.W., Sherman B.T., Lempicki R.A. (2009). Bioinformatics enrichment tools: Paths toward the comprehensive functional analysis of large gene lists. Nucleic Acids Res..

[167] Pomaznoy M., Ha B., Peters B. (2018). GOnet: A tool for interactive Gene Ontology analysis. BMC Bioinform..

[168] Okamura Y., Aoki Y., Obayashi T., Tadaka S., Ito S., Narise T., Kinoshita K. (2015). COXPRESdb in 2015: Coexpression database for animal species by DNA-microarray and RNAseq-based expression data with multiple quality assessment systems. Nucleic Acids Res..

[169] Okamura Y., Kinoshita K. (2018). Matataki: An ultrafast mRNA quantification method for large-scale reanalysis of RNA-Seq data. BMC Bioinform..

[170] Saitou N., Nei M. (1987). The neighbor-joining method: A new method for reconstructing phylogenetic trees. Mol. Biol. Evol..

[171] Tseng K.C., Li G.Z., Hung Y.C., Chow C.N., Wu N.Y., Chien Y.Y., Zheng H.Q., Lee T.Y., Kuo P.L., Chang S.B. (2020). EXPath 2.0: An Updated Database for Integrating High-Throughput Gene Expression Data with Biological Pathways. Plant Cell Physiol..

[172] Altschul S.F., Gish W., Miller W., Myers E.W., Lipman D.J. (1990). Basic local alignment search tool. J. Mol. Biol..

[173] Ogata Y., Suzuki H., Sakurai N., Shibata D. (2010). CoP: A database for characterizing co-expressed gene modules with biological information in plants. Bioinformatics.

[174] Ogata Y., Sakurai N., Suzuki H., Aoki K., Saito K., Shibata D. (2009). The prediction of local modular structures in a co-expression network based on gene expression datasets. Genome Inform..

[175] De Bodt S., Hollunder J., Nelissen H., Meulemeester N., Inze D. (2012). CORNET 2.0: Integrating plant coexpression, protein-protein interactions, regulatory interactions, gene associations and functional annotations. New Phytol..

[176] Zhang W., Morris Q.D., Chang R., Shai O., Bakowski M.A., Mitsakakis N., Mohammad N., Robinson M.D., Zirngibl R., Somogyi E. (2004). The functional landscape of mouse gene expression. J. Biol..

[177] Zhu Q., Wong A.K., Krishnan A., Aure M.R., Tadych A., Zhang R., Corney D.C., Greene C.S., Bongo L.A., Kristensen V.N. (2015). Targeted exploration and analysis of large cross-platform human transcriptomic compendia. Nat. Methods.

[178] Adler P., Kolde R., Kull M., Tkachenko A., Peterson H., Reimand J., Vilo J. (2009). Mining for coexpression across hundreds of datasets using novel rank aggregation and visualization methods. Genome Biol..

[179] Zoubarev A., Hamer K.M., Keshav K.D., McCarthy E.L., Santos J.R., Van Rossum T., McDonald C., Hall A., Wan X., Lim R. (2012). Gemma: A resource for the reuse, sharing and meta-analysis of expression profiling data. Bioinformatics.

[180] Szklarczyk D., Gable A.L., Nastou K.C., Lyon D., Kirsch R., Pyysalo S., Doncheva N.T., Legeay M., Fang T., Bork P. (2021). The STRING database in 2021: Customizable protein-protein networks, and functional characterization of user-uploaded gene/measurement sets. Nucleic Acids Res..

[181] Warde-Farley D., Donaldson S.L., Comes O., Zuberi K., Badrawi R., Chao P., Franz M., Grouios C., Kazi F., Lopes C.T. (2010). The GeneMANIA prediction server: Biological network integration for gene prioritization and predicting gene function. Nucleic Acids Res..

[182] Zuberi K., Franz M., Rodriguez H., Montojo J., Lopes C.T., Bader G.D., Morris Q. (2013). GeneMANIA prediction server 2013 update. Nucleic Acids Res..

[183] Raina P., Lopes I., Chatsirisupachai K., Farooq Z., de Magalhães J.P. (2021). GeneFriends 2021: Updated co-expression databases and tools for human and mouse genes and transcripts. bioRxiv.

[184] Miller H.E., Bishop A.J.R. (2021). Correlation AnalyzeR: Functional predictions from gene co-expression correlations. BMC Bioinform..

[185] Liberzon A., Subramanian A., Pinchback R., Thorvaldsdottir H., Tamayo P., Mesirov J.P. (2011). Molecular signatures database (MSigDB) 3.0. Bioinformatics.

[186] Wang P., Qi H., Song S., Li S., Huang N., Han W., Ma D. (2015). ImmuCo: A database of gene co-expression in immune cells. Nucleic Acids Res..

[187] Vandenbon A., Dinh V.H., Mikami N., Kitagawa Y., Teraguchi S., Ohkura N., Sakaguchi S. (2016). Immuno-Navigator, a batch-corrected coexpression database, reveals cell type-specific gene networks in the immune system. Proc. Natl. Acad. Sci. USA.

[188] Farber C.R., Mesner L.D., Rodriguez-Oquendo A. (2016). A Systems-Level Understanding of Cardiovascular Disease through Networks. Translational Cardiometabolic Genomic Medicine.

[189] Langfelder P., Zhang B., Horvath S. (2008). Defining clusters from a hierarchical cluster tree: The Dynamic Tree Cut package for R. Bioinformatics.

[190] Xu X., Lu X., Tang Z., Zhang X., Lei F., Hou L., Li M. (2021). Combined analysis of carotenoid metabolites and the transcriptome to reveal the molecular mechanism underlying fruit colouration in zucchini (*Cucurbita pepo* L.). Food Chem. Mol. Sci..

[191] Xie J., Ma A., Zhang Y., Liu B., Cao S., Wang C., Xu J., Zhang C., Ma Q. (2019). QUBIC2: A novel and robust biclustering algorithm for analyses and interpretation of large-scale RNA-Seq data. Bioinformatics.

[192] Hochreiter S., Bodenhofer U., Heusel M., Mayr A., Mitterecker A., Kasim A., Khamiakova T., Van Sanden S., Lin D., Talloen W. (2010). FABIA: Factor analysis for bicluster acquisition. Bioinformatics.

[193] Bergmann S., Ihmels J., Barkai N. (2003). Iterative signature algorithm for the analysis of large-scale gene expression data. Phys. Rev. E.

[194] Pontes B., Giráldez R., Aguilar-Ruiz J.S. (2015). Biclustering on expression data: A review. J. Biomed. Inform..

[195] Barrett T., Suzek T.O., Troup D.B., Wilhite S.E., Ngau W.C., Ledoux P., Rudnev D., Lash A.E., Fujibuchi W., Edgar R. (2005). NCBI GEO: Mining millions of expression profiles—Database and tools. Nucleic Acids Res..

[196] Russo P.S.T., Ferreira G.R., Cardozo L.E., Burger M.C., Arias-Carrasco R., Maruyama S.R., Hirata T.D.C., Lima D.S., Passos F.M., Fukutani K.F. (2018). CEMiTool: A Bioconductor package for performing comprehensive modular co-expression analyses. BMC Bioinform..

[197] Cardozo L.E., Russo P.S.T., Gomes-Correia B., Araujo-Pereira M., Sepulveda-Hermosilla G., Maracaja-Coutinho V., Nakaya H.I. (2019). webCEMiTool: Co-expression Modular Analysis Made Easy. Front. Genet..

[198] Vivian Li W., Li Y. (2021). scLink: Inferring Sparse Gene Co-expression Networks from Single-cell Expression Data. Genom. Proteom. Bioinform..

[199] Haas B.J., Papanicolaou A., Yassour M., Grabherr M., Blood P.D., Bowden J., Couger M.B., Eccles D., Li B., Lieber M. (2013). De novo transcript sequence reconstruction from RNA-seq using the Trinity platform for reference generation and analysis. Nat. Protoc..

[200] Bryant D.M., Johnson K., DiTommaso T., Tickle T., Couger M.B., Payzin-Dogru D., Lee T.J., Leigh N.D., Kuo T.H., Davis F.G. (2017). A Tissue-Mapped Axolotl De Novo Transcriptome Enables Identification of Limb Regeneration Factors. Cell Rep..

[201] Haque A., Engel J., Teichmann S.A., Lonnberg T. (2017). A practical guide to single-cell RNA-sequencing for biomedical research and clinical applications. Genome Med..

[202] Moll P., Ante M., Seitz A., Reda T. (2014). QuantSeq 3′ mRNA sequencing for RNA quantification. Nat. Methods.

[203] Corley S.M., Troy N.M., Bosco A., Wilkins M.R. (2019). QuantSeq. 3′ Sequencing combined with Salmon provides a fast, reliable approach for high throughput RNA expression analysis. Sci. Rep..

[204] Logotheti S., Pavlopoulou A., Galtsidis S., Vojtesek B., Zoumpourlis V. (2013). Functions, divergence and clinical value of TAp73 isoforms in cancer. Cancer Metastasis Rev..

[205] Policastro R.A., Zentner G.E. (2021). Global approaches for profiling transcription initiation. Cell Rep. Methods.

[206] Policastro R.A., Zentner G.E. (2022). Genome-Wide Profiling of Transcription Initiation with STRIPE-seq. Methods Mol. Biol..

[207] Cole C., Byrne A., Beaudin A.E., Forsberg E.C., Vollmers C. (2018). Tn5Prime, a Tn5 based 5′ capture method for single cell RNA-seq. Nucleic Acids Res..

[208] Picelli S., Faridani O.R., Bjorklund A.K., Winberg G., Sagasser S., Sandberg R. (2014). Full-length RNA-seq from single cells using Smart-seq2. Nat. Protoc..

[209] Ostlund G., Sonnhammer E.L. (2014). Avoiding pitfalls in gene (co)expression meta-analysis. Genomics.

[210] Michiels S., Koscielny S., Hill C. (2005). Prediction of cancer outcome with microarrays: A multiple random validation strategy. Lancet.

[211] Huang J., Vendramin S., Shi L., McGinnis K.M. (2017). Construction and Optimization of a Large Gene Coexpression Network in Maize Using RNA-Seq Data. Plant. Physiol..

